# In silico identification and ex vivo evaluation of *Toxoplasma gondii* peptides restricted to HLA-A*02, HLA-A*24 and HLA-B*35 alleles in human PBMC from a Colombian population

**DOI:** 10.1007/s00430-024-00815-x

**Published:** 2024-12-31

**Authors:** Mónica Vargas-Montes, María Camila Valencia-Jaramillo, Juan David Valencia-Hernández, Jorge Enrique Gómez-Marín, Ailan Farid Arenas, Néstor Cardona

**Affiliations:** 1 Grupo de Estudio en Parasitología Molecular (GEPAMOL), Faculty of Health Sciences, Centro de Investigaciones Biomédicas, Universidad del Quindío, Quindio, Armenia, Colombia; 2 Faculty of Dentistry, Universidad Antonio Nariño, Quindio, Armenia, Colombia

**Keywords:** Peptides, Artificial neural networks, HLA-I alleles, IFN-γ, CD107a

## Abstract

*Toxoplasma gondii* infects approximately 30% of the population, and there is currently no approved vaccine. Identifying immunogenic peptides with high affinity to different HLA molecules is a promising vaccine strategy. This study used an in silico approach using artificial neural networks to identify *T. gondii* peptides restricted to HLA-A*02, HLA-A*24, and HLA-B*35 alleles. Proteomes from seven *T. gondii* strains and transcriptomic data of overexpressed genes from *T. gondii-*RH in human PBMC were also used. Parasite protein sequences were analyzed with R *‘Epitope Prediction’* library. Peptide candidates were evaluated in the artificial neural networks based on the probabilities of output neurons (*p* > 0.5). The IFN-γ responses in PBMC from *T. gondii* seronegative and seropositive individuals were evaluated by ELISpot. Peptides with higher IFN-γ induction were evaluated to identify cytotoxic response in CD8^+^ T cells (CD107a). In silico analysis identified 36 peptides from *T. gondii* proteins with predicted affinity to HLA-A*02, A*24, and B*35 alleles. Experiments with PBMCs revealed that a peptide restricted to HLA-A02 (P1: FLFAWITYV) induced a significant increase in IFN-γ-producing cells (*p* = 0.004). For HLA-A24, a peptide (P8: VFAFAFAFFLI) also induced a significant IFN-γ response (*p* = 0.004), while for the HLA-B*35 allele, the P6 peptide (YPIAPSFAM) induced a response that differed significantly from the control (*p* = 0.05). These peptides induced also a significant percentage of central memory CD8 + T cells expressing the degranulation marker CD107a (*p* < 0.05). Finally, we identified three *T. gondii* peptides that induced IFN-γ response, and a cytotoxic response measured by CD107a expression on CD45RAneg-CD8 cells. These peptides could be considered part of a multi-epitope vaccine against toxoplasmosis in humans.

**Supplementary Information:**

The online version contains supplementary material available at 10.1007/s00430-024-00815-x.

## Introduction

Toxoplasmosis is a zoonotic infection caused by *Toxoplasma gondii* (*T. gondii*), an intracellular protozoan with a cosmopolitan distribution that infects most homeothermic animals, including birds, mammals, and humans [1]. Approximately one-third of the global human population has antibodies against *T. gondii*, indicating prior exposure to this parasite [2]. This infection can result in severe complications, particularly in immunocompromised individuals with HIV and cancer, transplant patients, pregnant women, and newborns [3–5]. Furthermore, *T. gondii* infection has been linked to various mental health disorders, including schizophrenia, bipolar disorder, and suicidal behavior [6]. Transmission of the parasite primarily occurs through the consumption of contaminated meat, vegetables, or water [7–9]. As a result, the Food and Agriculture Organization (FAO) and the World Health Organization (WHO) have classified toxoplasmosis as a foodborne disease of global concern [10].

Currently, there are no vaccines for toxoplasmosis in humans, and therapeutic options are limited in efficacy and accessibility [11]. Moreover, the available therapies for treating toxoplasmosis are not entirely safe or effective [12, 13]. To date, only one commercially available vaccine (*Toxovax™*) has been specifically authorized for use in sheep [14]. This vaccine, which is based on attenuated live tachyzoites from the S48 strain, is incapable of forming cysts or oocysts. Although *Toxovax* induces potent cellular immunity in sheep, it has significant limitations in terms of safety, production, and stability, rendering it unsuitable for use in humans [15]. In addition to live attenuated parasites, different approaches have been explored for toxoplasmosis vaccine development, including the preparation of total antigens, recombinant proteins, DNA vaccines, and carbohydrates [16]. However, these strategies have been insufficient for identifying vaccine candidates capable of generating a long-term protective immune response against the parasite and effectively eliminating tissue cysts [17].

In the search for toxoplasmosis vaccine candidates in humans, the use of immunogenic peptides has also been reported as a low-cost, efficient, and highly precise strategy [18]. These subunit vaccines offer the advantage that specific antigens can be processed without the presence of additional antigens that may be irrelevant. Furthermore, a moderate immune response induced by parasite antigens can be enhanced using adjuvants [19]. Currently, there are ongoing studies on infectious diseases such as malaria, influenza, COVID-19, and HIV, reporting the use of peptide-based vaccines in phase I, II, and III clinical trials [20].

An effective vaccine against toxoplasmosis is suggested to be based on the proposed model of protective immunity, dependent on the activation of the cellular immune response (Th1) with cytotoxic CD8 + T lymphocytes [21] and efficient induction of specific CD4 + T cells, both of which participate in protection through the secretion of effector and inflammatory cytokines such as IFN-γ, TNF-α, IL-1, and IL-6 [22]. Specifically, CD8 + T cells recognize octameric/nonameric peptides, or even longer sequences [23], which are presented by major histocompatibility complex I molecules (HLA-I in humans) on infected cells and are crucial for the activation of the immune response and subsequent parasite clearance [24]. Therefore, an approach for the development of an efficient vaccine against *T. gondii* should include epitopes that stimulate and enhance host cellular immunity, specifically restricted to the most prevalent HLA complexes in the human population.

These peptide sequences or epitopes should be identified from proteins present across different stages and strains of the parasite, with high immunogenic potential and high expression levels [25]. This strategy, framed within reverse vaccinology [26], allows for the selection of vaccine candidates considering the parasite’s proteomic diversity, antigenic variation, and the search for recognition by the majority of the human population. This is achieved using bioinformatics tools and the available biological information (genomes and proteomes) [25, 26]. Recent advancements have been crucial in determining which antigens are most likely to induce a protective immune response across the majority of the human population, particularly if the peptides are recognized by super-HLA haplotypes present in 80–90% of the human population [27].

Considering the above, a previous study performed by the GEPAMOL research group in Colombia found that peptides from *T. gondii* with high affinity for the HLA-A*02 supertype (present in approximately 40–50% of the world’s human population) can be selected using a bioinformatics approach [28]. When tested on peripheral blood mononuclear cells (PBMC) from individuals with chronic-asymptomatic toxoplasmosis and HLA-A*02 positivity, these peptides activated specific cellular responses measured in the presence of IFN-γ [28]. Similar studies have been previously reported, in which bioinformatic analysis identified peptides derived from *T. gondii* secretory and surface proteins with high affinity for HLA-A*02, HLA-A*11, and HLA-B*07 haplotypes, which induce an IFN-γ response in human CD8 + T cells and transgenic mice with these supertypes [29–31]. These studies further demonstrated that the use of computational tools constitutes a useful and valuable strategy for selecting potential vaccine candidates.

Currently, the in silico discovery of potential vaccine candidates is considered a powerful approach that saves time and resources by reducing the number of candidates for experimental testing [32, 33]. Similarly, in recent years, numerous bioinformatics advances, led by artificial intelligence, have generated a paradigm shift towards high-performance in silico methods for the selection and discovery of new promising vaccine candidates [32].

Given the previous reasons and considering that peptides previously evaluated for *T. gondii* have been restricted to a limited number of HLA-I haplotypes and are derived from a select group of secretory and surface proteins of the parasite, it is important to identify new immunogenic epitopes. Utilizing in silico analysis to select new antigens from the entire *T. gondii* proteome that exhibit immunogenic characteristics and high affinity for other HLA-I frequent haplotypes such as HLA-A*24 and HLA-B*35 is important and necessary. These haplotypes, in addition to HLA-A*02, would cover a large part of the global human population (~ 70%) [34–39]. Therefore, the aim of this study was to develop an in silico strategy for the identification of *T. gondii* peptides restricted to three HLA-I (HLA-A*02, HLA-A*24, and HLA-B*35) and to perform ex vivo evaluation of PBMC from individuals with chronic-asymptomatic toxoplasmosis. These newly identified peptides could be a part of a multi-epitope vaccine model against toxoplasmosis in humans.

## Materials and methods

**Fig. 1:**
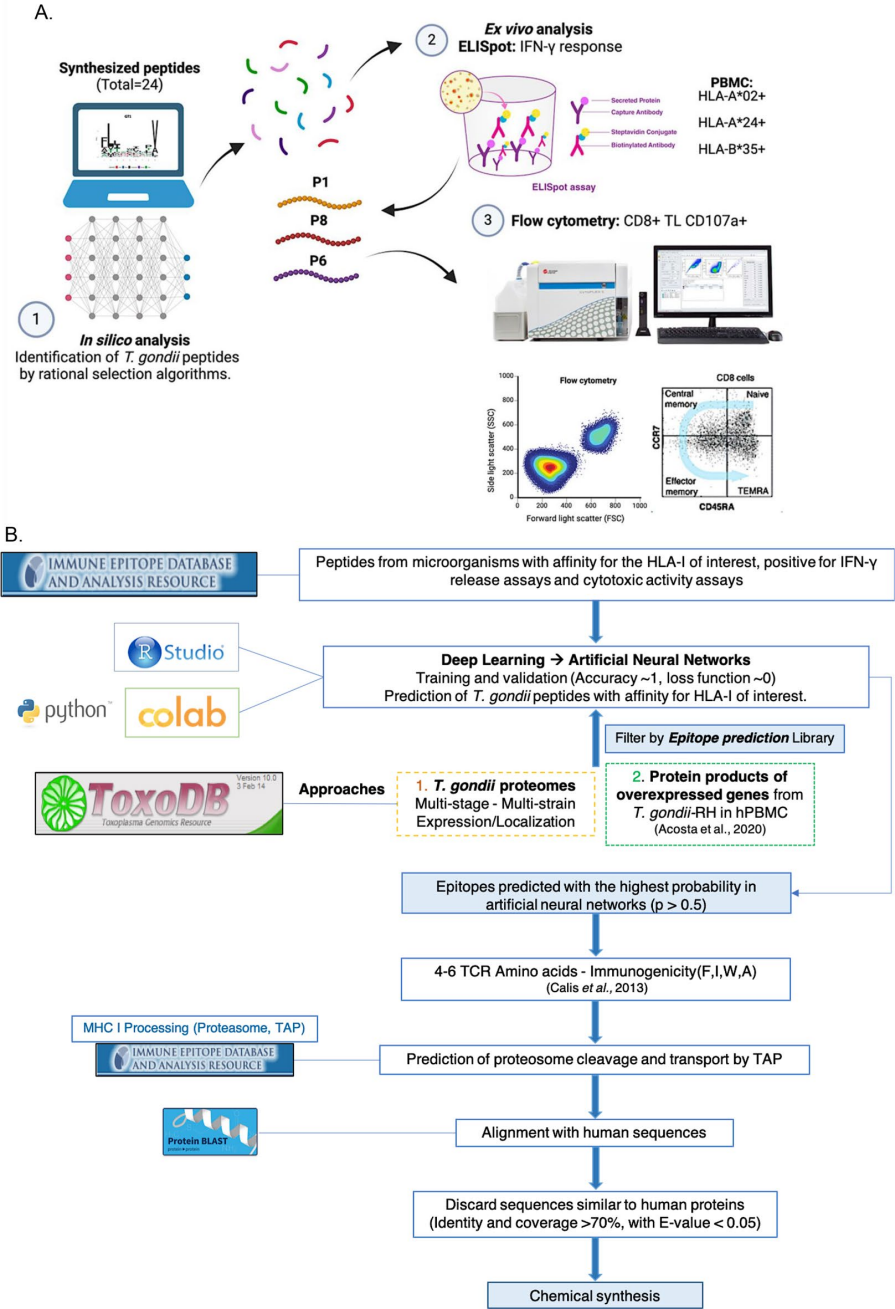
(**A**) Schematic representation of the general methodology used in the study, including a first in silico phase for the selection of the *T. gondii* peptides and a second laboratory (ex vivo) phase for the validation and evaluation of the peptide sequences using human PBMC with the specific HLA-I alleles. Created with BioRender.com. (**B**) Flowchart summarizing the in silico methodology developed for the selection of *T. gondii* peptides

The general methodology used in this work included the first in silico phase for the selection of *T. gondii* peptides restricted to HLA-I haplotypes and a second laboratory (ex vivo) phase for the validation and evaluation of the peptide sequences using human PBMC with specific HLA-I alleles. A schematic representation of the general methodology is shown in Fig. 1A.

### In silico analysis

#### Training and validation of artificial neural networks

An artificial intelligence method based on deep learning was employed, and the architecture of artificial neural networks was applied. RStudio [40] (R 3.5.3) and the Python-Colab platform (https://colab.research.google.com/) [41] were used to construct the neural network models. Additionally, the Keras library was used to implement the deep learning models. Specifically, feedforward neural networks and long short-term memory (LSTM) networks have been explored [42], which have been used to predict anti-cancer peptides [43]. Peptide sequences of 9 and 10 amino acids (AA) for network training were obtained from the IEDB database (http://www.iedb.org/) [44], including peptides from all reported microorganisms with affinity for the HLA-I of interest and other globally frequent HLA-A and B (18 alleles in total, frequency > 1% according to the Allele Frequency Net database, (http://www.allelefrequencies.net/), and corresponding to peptides experimentally evaluated in cytotoxic activity assays. This was performed to obtain a more enriched training dataset for the development and validation of neural networks. The peptides used for network training were classified as positive or negative, according to the experimental results reported in the IEDB database, and 20% of the total sequences were used for model validation. Different network architectures were evaluated by changing the number of layers and neurons until a loss function close to zero and an accuracy close to one were reached. Therefore, the validation metrics of the models include the loss function (~ 0) and accuracy (~ 1) [45].

#### Prediction of epitopes from T. gondii protein sequences

Once the neural network models were established, *T. gondii* protein sequences were determined, and epitope prediction was performed. Two approaches were employed: Approach 1, using the complete *T. gondii* proteomes available in the ToxoDB database; and Approach 2, taking advantage of the availability of transcriptomic data from the GEPAMOL research group, specifically selecting the protein products from overexpressed genes of the parasite during infection of human PBMC, which have a high probability of entering the MHC-I processing pathway, according to theory [46].

Approach 1 was performed using the proteomes of 7 *T. gondii* strains obtained from the ToxoDB v.46 database (https://toxodb.org/toxo/) including the strains GT1 (type I), ME49 (type II), VEG (type III), ARI (atypical, USA), MAS (atypical, France), VAND (atypical, French Guiana), CATBr9 (atypical, Brazil). These strains represent the three clonal lineages of the parasite, atypical strains, and different geographical origins. For the second approach, we used data derived from a doctoral research project [47], including only the protein products from genes with the highest expression level of the *T. gondii* RH strain after 1 h of infection to human PBMC, comprising a total of 16 protein sequences.

The protein sequences from both approaches were analyzed using an initial filter with the R library *‘Epitope Prediction*’ [48], predicting epitopes of 9 and 10 AA, with an IC50 < 2nM for approach 1 and IC50 < 50nM for approach 2, and with affinity for all HLA-I alleles of interest. The pre-selected sequences were then evaluated in the trained artificial neural networks, classifying sequences with probabilities greater than 0.5 (*p* > 0.5) as positive. Sequence logos were generated using the R *PepTools* library for peptides with the highest probability (*p* > 0.90). After selecting the peptides with the highest probabilities and those that were redundant among parasite strains, we described the proteins from which epitopes were derived using the information available in ToxoDB. This included their expression at different stages of the parasite (oocyst, bradyzoite, and tachyzoite, with RMA values > 3) and their subcellular localization.

As the final criteria for the bioinformatics analysis, the residues in positions 4–6 of each peptide were analyzed, as these have been described as important for TCR interaction and T cell activation [49]. Additionally, we predicted proteasome cleavage and TAP transport using the Proteasomal cleavage/TAPtransport/MHCclass-I combined predictor server from IEDB (http://tools.iedb.org/processing/). Finally, peptide sequences were aligned using the BLASTp tool (https://blast.ncbi.nlm.nih.gov/Blast.cgi?PAGE=Proteins) from NCBI to exclude sequences similar to human proteins (identity and coverage > 70% and an E value < 0.05). A graphical summary of the in silico methodology is presented in Fig. 1B. Most of the predicted *T. gondii* peptide epitopes with the best scores after all in silico selection processes were chemically synthesized by Peptide 2.0 (Chantilly, Virginia, USA).

### Ex vivo analysis of the T. gondii peptides

#### Selection of the population included in the study

All participants involved in the study were volunteers who provided written informed consent prior to blood sample collection. This study was conducted in accordance with the Declaration of Helsinki, which considers the ethical principles for medical research involving human subjects. All individuals agreed to participate in the study and signed an informed consent form according to resolution 8430 of the Ministry of Health of Colombia [50]. A total of fifty individuals (*n* = 50) between 18 and 50 years old were included in this study and were recruited at the Biomedical Research Center (CIBM) of the University of Quindío, Colombia.

Two peripheral venous blood samples were collected from each individual: the first (8 mL of blood) for serology (IgM and IgG anti-*Toxoplasma* antibodies) and DNA isolation to analyze the presence of HLA-I alleles. For the second sample (20 ml of whole blood), a subsample of approximately 10 individuals positive for each allele of interest was selected (five seronegative and five seropositive, with chronic-asymptomatic toxoplasmosis) to isolate PBMC and to evaluate the immune response. These individuals were diagnosed with toxoplasmosis by an expert physician at the Health Center of the University of Quindío. Serology of each sample was performed using a commercial ELFA with VIDAS TOXO IgG II (Ref. 30210) and TOXO IgM (Ref. 30202) kit (BioMérieux) [51, 52].

#### DNA extraction and typing of HLA-I alleles

To identify individuals positive for the alleles of interest, DNA was isolated using the GenElute kit (Ref. G1N701KT, SIGMA) according to the manufacturer’s instructions. DNA samples were then stored at -80 °C for subsequent analysis. PCR assays were performed using allele-specific primers. The sequences of the primers used, and length of the expected PCR products are summarized in Table 1. The primers used for each allele were selected from the literature [53–55] or designed using different web tools (Primer BLAST, Primer 3, and OligoAnalyzer). The PCR amplification was performed using GoTaq^®^ Green Master Mix (Promega. Reference M7122), with primers at a final concentration of 0.4 µM, and 100 ng of DNA per sample.

**Table 1:** Sequences of the primers used and size (base pairs) of the expected PCR products for each HLA-I gen are summarized in the table

HLA allele	Forward primer (5’-3’)	Reverse primer (5’-3’)	Expected product size
HLA-A*02	TCCTCGTCCCCAGGCTCT	CCAGGTAGGCTCTCAACTGC	734 pb
HLA-A*24	ACTGACCGAGAGAACCTGCGGAT	ACTTGCGCTTGGTGATCTGAGCC	464 pb
HLA-B*35	GCCGCGAGTCCGAGGAC	GCCATACATCCTCTGGATGA	428 pb

Allele-specific amplification cycles were conducted as follows: For HLA-A*02, an initial denaturation was performed at 95 °C for 5 min, followed by 25 cycles at 95 °C for 35 s, 60 °C for 30 s, and 72 °C for 55 s, with a final extension at 72 °C for 10 min. For HLA-A*24, the methodology proposed by Nakatsugawa et al. [54] was employed, with minor modifications, starting with an initial denaturation at 94 °C for 2 min, followed by 35 cycles at 94 °C for 15 s, 68 °C for 30 s, and 72 °C for 30 s, with a final extension at 72 °C for 5 min. Finally, for HLA-B*35, initial denaturation was performed at 94 °C for 2 min, followed by 30 cycles at 94 °C for 30 s, 58 °C for 30 s, and 72 °C for 30 s, with a final extension at 72 °C for 5 min. Amplification was performed using a SimpliAmp thermal cycler (Applied Biosystems). Amplicons from all samples were visualized on 1.5% agarose gels with electrophoretic runs at 50–100 V for 45 min to 1 h.

#### PBMC isolation and evaluation of IFN-γ response

A second blood sample was collected from 10 individuals previously classified as HLA-A*02+, 10 HLA-A*24 + individuals, and 6 HLA-B*35 + for PBMC isolation and evaluation of the peptide-stimulated IFN-γ response. PBMC extraction was carried out by density gradient centrifugation using Histopaque^®^-1077 reagent (Sigma-Aldrich, Darmstadt, Germany) following the manufacturer’s instructions. Once isolated, the cells were resuspended in RPMI-1640 medium (Gibco), and cell counting was performed using trypan blue and a Neubauer chamber. The isolated PBMC were immediately used in IFN-γ measurement experiments and/or cryopreserved in RPMI supplemented with 10% inactivated fetal bovine serum (Fetal Bovine Serum, Gibco, United States), 1% penicillin/streptomycin (Pen Strep Penicillin Streptomycin, Reference 15140122, Gibco, United States), and 10% dimethyl sulfoxide (DMSO) for storage at -80 °C and later use.

IFN-γ ELISpot assays were performed as previously reported [56] using the Mabtech kit (Cincinnati, OH) to evaluate IFN-γ production in isolated PBMC. Initially, the 96-well plate (MSIPS4W10 Multiscreen HTS-IP, Millipore, Bedford, MA, USA) was humidified with 20 µL of 35% ethanol, followed by five washes with sterile water. The wells were then coated by adding 15 µg/ml of anti-IFN-γ capture monoclonal antibody (1-D1K, Mabtech) diluted in sterile 1X PBS (Phosphate Buffered Saline, pH 7.4, Ref. 70011044, Gibco) at a volume of 50 µL per well and incubated overnight at 4 °C. The next day, the plate was washed five times with 1X PBS and blocked with RPMI-1640 and 10% fetal bovine serum for two hours at room temperature. Subsequently, PBMC samples were added to the plate in supplemented RPMI, adding 250,000 cells per well with the following stimuli in triplicates. Selected peptides restricted to HLA-A*02, A*24, and B *35 molecules (previously resuspended in DMSO) were added at a final concentration of 20 µg/ml and diluted in RPMI-1640 medium (DMSO concentration of 0.2% per well). Positive controls included Phorbol Myristate Acetate (PMA) at 5 ng/ml, Ionomycin at 0.5 µg/ml (Sigma, C27275), and total soluble extract of *T. gondii* RH strain at 10 µg/ml. Additionally, two positive peptide controls were included, P28 (FMGVLVNSL) and P29 (FLDRALLTL), from the *T. gondii* proteins GRA6 and Got1 [28, 57] specific for the HLA-A*02 allele. Finally, as negative controls, a vehicle control with 0.2% DMSO diluted in RPMI, a cell control without stimulus (CC), and an irrelevant peptide control (YPHFMPTNL) from murine cytomegalovirus phosphoprotein PP89 (YL9) were used.

The stimulated PBMC were incubated for 48 h with the respective stimuli at 37 °C and 5% CO2. After incubation, the plate was washed five times, and 100 µL/well of biotinylated anti-IFN-γ detection antibody (7B6-1, Mabtech) at 1 µg/ml was added for two hours at room temperature. The plate was then washed again, and 100 µL of streptavidin-conjugated alkaline phosphatase (1:1000) was added and incubated for one hour at room temperature. Subsequently, the plate was washed five times, and spots were developed by adding 100 µL of BCIP/NBT substrate (Mabtech). Spot development was monitored for approximately 10 min and the reaction was stopped by thorough washing with distilled water. Finally, the plates were air-dried and stored overnight at 4 °C in the dark. To calculate the number of IFN-γ-producing cells, each well was photographed using an 18.0 megapixel digital camera with a stereoscope (ZEIZZ Stemi DV4), and the spots were counted using ImageJ software (http://imagej.nih.gov/ij). The results are expressed as the number of spot-forming cells (SFCs). The stimulation index (SI) for each peptide was calculated as previously reported [56]. SI was considered significant when the value was greater than 2, where *stimulation index = x̄ SFC stimulus/x̄ DMSO control.*

#### Evaluation of the activation of cytotoxic mechanism activation (CD107a) in CD8 + T lymphocytes

To determine whether the selected peptides induced the activation of effector mechanisms in memory CD8 + T lymphocytes (TL), the presence of the degranulation marker CD107a (lysosome-associated membrane protein, LAMP-1) was evaluated. Thirteen samples from *T. gondii* seropositive individuals (five for HLA-A*02, five for HLA-A*24, and three for HLA-B*35) and nine seronegative individuals (three for each HLA-I) were included. Cryopreserved PBMC were thawed in water at 37 °C, diluted in pre-warmed supplemented RPMI (10% fetal bovine serum, 1% pen/strep), and washed at 300 RCF for 10 min. After washing, cells were resuspended in supplemented RPMI and plated on 24-well plates, adding 1 × 10^6^ PBMC/well with the respective stimuli: peptides for HLA-A*02, A*24, and B*35 (previously selected by ELISpot as IFN-γ inducers) at 20 µg/ml. PMA (5 ng/ml) and Ionomycin (0.5 µg/ml, and the total extract of *T. gondii* at 10 µg/ml was used as a positive control. Finally, as negative controls, we used the vehicle control (0.2% DMSO) and an irrelevant peptide control (YL9).

The anti-CD107a antibody was added at the beginning of the stimulation period (anti-human CD107a-FITC, BioLegend, Clone: H4A3), as previously described [58]. 1 h post-stimulation, monensin at 1 µg/ml (1X, BioLegend) was added to prevent the reinternalization of CD107a. The cells were then incubated for 6 h at 37 °C and 5% CO_2_. After incubation, the PBMC were collected and washed with 1X PBS by centrifugation at 300 RCF for 5 min. The cell pellet was resuspended in 100 µL of staining buffer (1X PBS supplemented with 0.1% NaN_3_ and 0.5% BSA) and a cocktail of antibodies was added to stain surface markers of the target cell population: anti-human CD3-Pe-Dazzle (BioLegend, Clone: SK7), anti-human CD8-PE/Cy7 (BioLegend, Clone: HIT8a), anti-human CD45RA-APC/Cy7 (BioLegend, Clone: HI100), and anti-human CCR7-BV421 (BioLegend, Clone: G043H7). The cells were then incubated on ice for 20 min in the dark. Next, the PBMC were washed again, and the stained cells were resuspended in 500 µL of acquisition buffer (ISOFLOW, EPICS SHEATH, ref. 854685910, Beckman Coulter). Finally, the viability cell marker 7AAD (Viability Staining Solution, reference 420404, BioLegend) was added and the cells were incubated for 5 min on ice in the dark. The stained cells were transferred to flow cytometry tubes (12 × 75 mm), and acquisition was performed using a CytoFLEX S flow cytometer (Beckman Coulter).

At least 100,000 events were acquired for each sample using the stimuli and controls. Beads (Versa Comp Antibody Capture Bead Kit). Reference: B22804) was employed to compensate for the fluorescence channels used in the experiment. CytExpert software was used for data processing and analysis. An initial gate was set for the lymphocyte population and a subsequent gate was established to remove the doublets. In singlet events, a quadrant for viable cells was defined, followed by gates for CD3 + CD8 + cells, CD45RAneg/CCR7neg (effector memory), and CD45RAneg/CCR7pos (central memory). In these populations, the positivity threshold for the CD107a marker was established according to the quadrant set for unstimulated samples (with vehicle control, DMSO 0.2%).

### Statistical analysis

Data are presented as mean and standard deviation (SD) or median and interquartile range (IQR), depending on their distribution. Specifically, ELISpot results are presented as median and IQR values due to the non-normal distribution of the data and its ability to provide a robust measure of central tendency and variability. The Shapiro-Wilk test was used to assess normality. Kruskal-Wallis test with Dunn’s multiple comparisons test, or the Mann-Whitney U test, was employed to compare the stimulus vs. the vehicle control. All analyses were performed using GraphPad Prism software (version 9.0; San Diego, CA, USA), and graphs were generated using this software. Statistical significance was set at p value ≤ 0.05.

## Results

### In silico analysis: neural networks training and validation

**Fig. 2:**
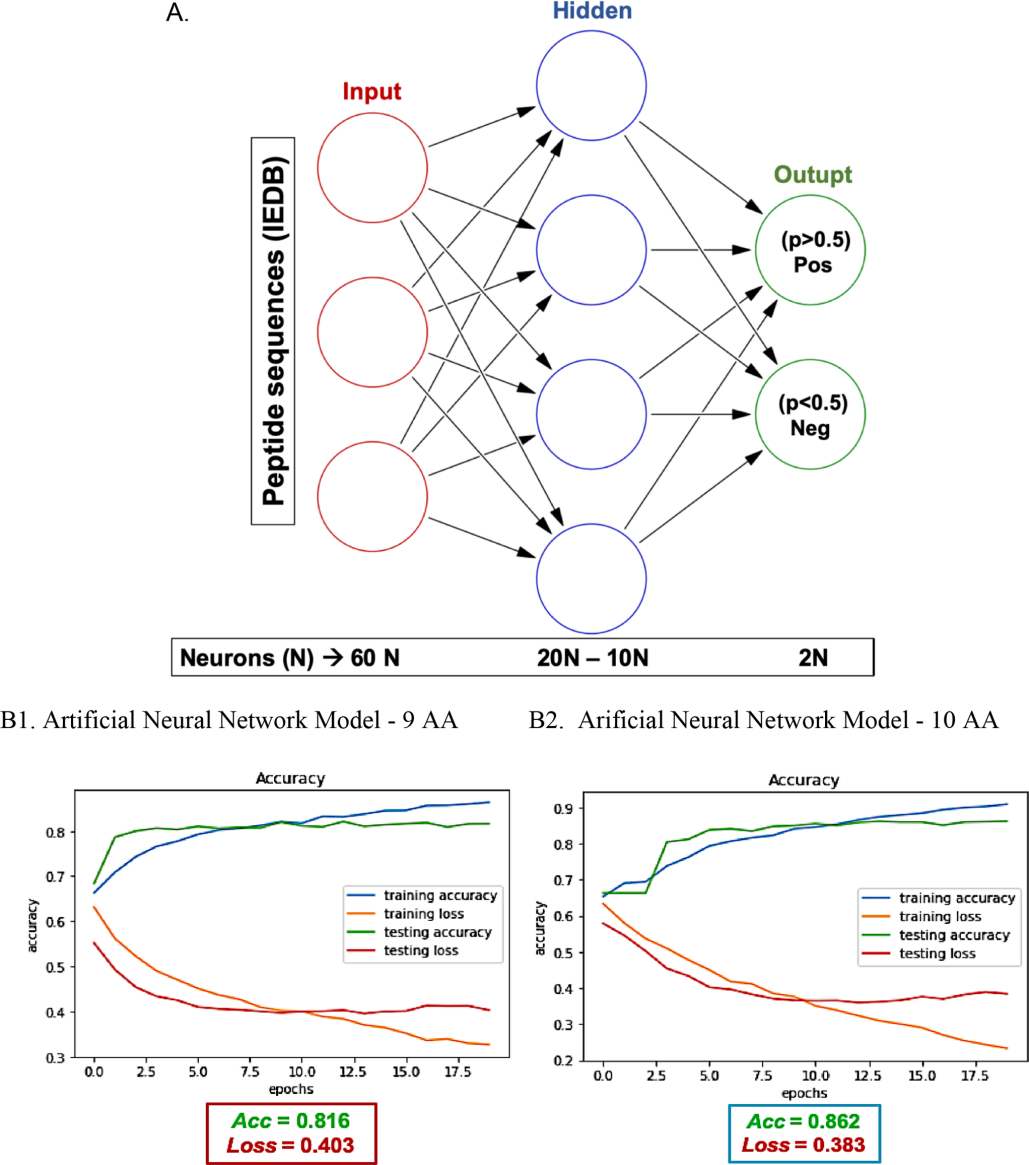
(**A**) Visualization and (**B**) validation of artificial neural network architectures with two output neurons trained to predict peptide sequences likely to interact with MHC class I molecules of interest. The accuracy and loss function values of the selected models are shown in red the red and blue boxes. (**B1**) Validation for 9 AA neural network model. (**B2**) Validation for 10 AA neural network model. In each plot the accuracy is represented by green lines and loss by the red lines. Blue and orange lines correspond to the training set and show a similar behavior to the testing set

A total of 13,468 peptide sequences of 9 amino acids (AA) from the IEDB database were included for training the first neural network, including peptides from all reported microorganisms with affinity for the HLA-I of interest (HLA-A*02, HLA-A*24, and HLA-B*35) and other globally frequent HLA-A and B. Over 30 different architectures were evaluated for the new neural network (by varying the number of neurons and layers), and the 60-20-10-2 (neurons/layer) architecture (Fig. 2A) achieved the most suitable metrics for the validation set, obtaining values of 0.40 and 0.82 for loss and accuracy respectively (Fig. 2B1), indicating that there was no overfitting in the model [59]. For the second neural network, with peptides of 10 AA in length, a total of 4,596 sequences were included, and a similar architecture to that used for the nine AA peptides was employed, obtaining an accuracy of 0.86 and a loss of 0.38 (Fig. 2B2). With these validated models for neural networks, the evaluation of *T. gondii* protein sequences were the next step to predict the most probable peptide epitopes that could interact with the different HLA-I of interest.

### Identification of T. gondii peptides

**Fig. 3:**
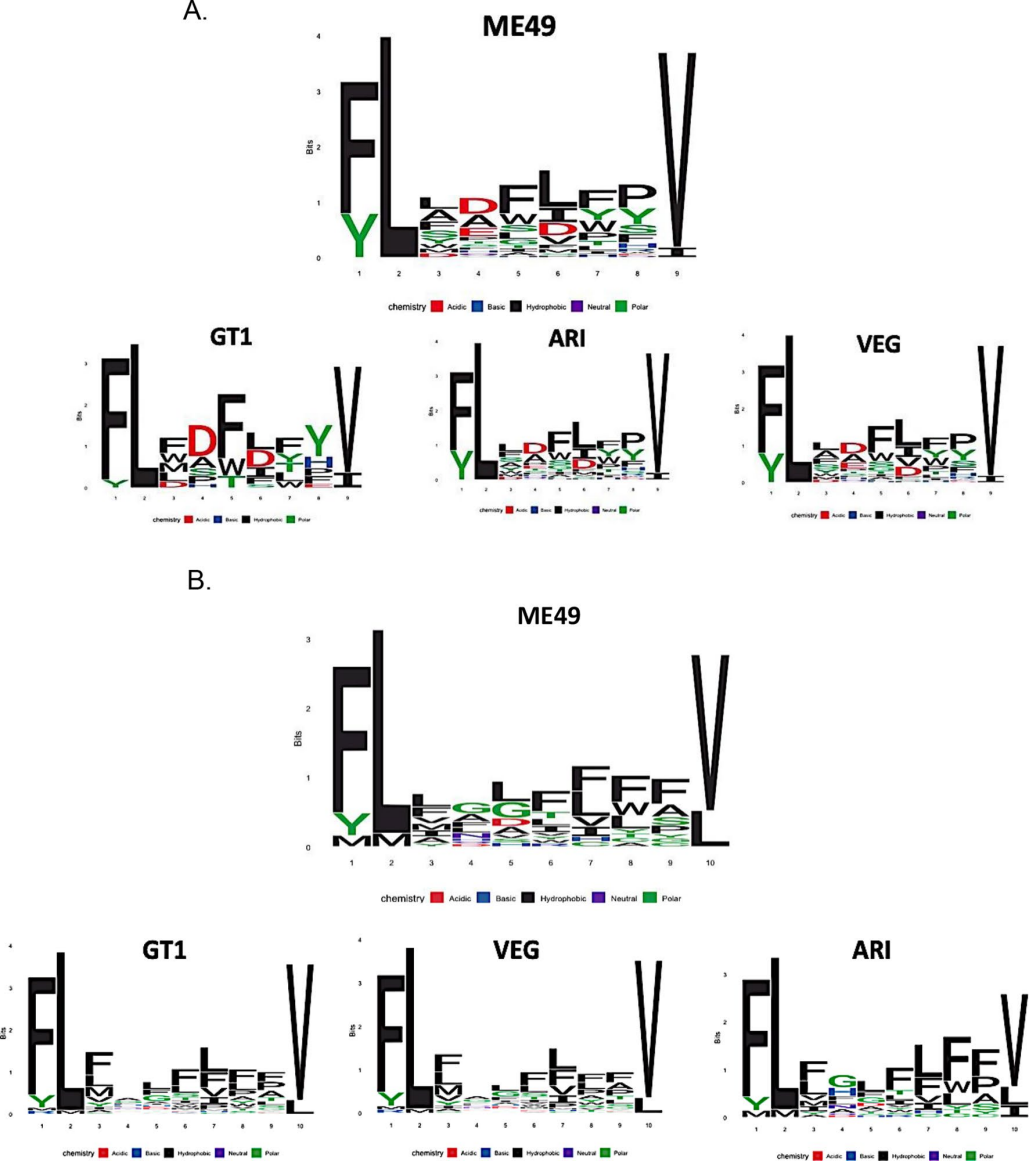
Peptide logos. (**A**) 9-AA peptide logos predicted by the neural network with higher probability (*p* > 0.998) in approach 1. Logo for strain ME49 and 3 different *T. gondii* strains are shown as examples. (**B**) Logos of 10 AA peptides predicted by the neural network with higher probability (*p* > 0.95) in approach 1. Logo for strain ME49 and 3 different *T. gondii* strains are shown as examples. Sequence logos were generated using the R *Peptools* library

Regarding approach 1, using the complete *T. gondii* proteomes from 7 different parasite strains (GT1, ME49, VEG, ARI, MAS, VAND, CATBr9), between 5,000 and 5,450 9-AA peptide epitopes were predicted for each strain using “*Epitope prediction*” R library. These predicted epitopes were analyzed using the neural network trained for 9-AA peptides, identifying a total of 16–40 peptides with the highest probabilities (*p* > 0.998) across the different strains. These peptides were visualized through sequence logos, revealing frequent patterns of leucine (L) and valine (V) at positions 2 and 9, respectively (Fig. 3A), corresponding to hydrophobic amino acids [60]. Considering the analysis of proteomes for 10-AA peptides, between 600 and 685 10-mer epitopes were predicted for each of the 7 proteomes examined using “*Epitope prediction*” tools. Evaluation of these epitopes using the neural network also identified 15–40 peptides per strain with the highest probabilities (*p* > 0.95). Visualization of these sequences again exhibited a pattern of hydrophobic residues (F and L) at positions 1 and 2 and V/L at the C-terminal position (Fig. 3B).

In the group of 9-AA peptide sequences with the highest probabilities, a total of six peptides were identified as redundant for the seven strains of *T. gondii*, which exclusively bound to HLA-A*02 (Supplementary Table 1, St1). For HLA-B*35, a lower cut-off point (*p* > 0.80) was chosen, resulting in the identification of three redundant peptides restricted to B*35 across the strains (St1). For the HLA-A*24 supertype, no epitopes of this size were predicted with the initial cut-off value set in *Epitope Prediction* (IC50 < 2nM); therefore, an additional prediction for this supertype was necessary with a higher cut-off (IC50 < 50nM) (St 2). The 9-mer peptides identified for A*02 and B*35 in approach 1 were derived from a diverse array of parasite proteins, including membrane proteins, secretory organelle proteins, such as rhoptries and micronemes, cytosolic proteins, and several hypothetical proteins (St1). All of these proteins corresponded to genes expressed in the three stages of the parasite (oocyst, tachyzoite, and bradyzoite) according to ToxoDB (St1). Regarding the analysis of amino acids located at positions 4–6 [49], in the 9-mer peptides identified by the network, the majority exhibited residues positively associated with immunogenicity (8/9 peptides). In addition to the previous analysis, all peptides were found to have a positive cleavage prediction for proteasome/TAP (St1). Finally, the alignment performed with BLASTp indicated that peptide No.3 (FLLDFLLYV) from the RON3 protein was the only peptide with an identity greater than 70% with a human protein; therefore, this peptide was excluded from the final selection. To this point, eight 9-mer peptides restricted to A*02 and B*35 were filtered using approach 1 (St1).

Considering HLA-A*24 supertype, the analysis performed in ‘*Epitope Prediction*,’ increasing the IC50 criterion to 50 nM for proteome evaluation, generated a total of ~ 700 9-mer epitopes per strain, as well as 3,300-3,400 10-mer epitopes binding to the A*24 supertype, which were analyzed using neural networks. The four 9-mer peptides were redundant across strains and showed the highest probability (*p* > 0.70) (St2). Regarding the 10-mer epitopes, only one peptide (RWFWTFRFNW) from a hypothetical protein was classified as positive by the network and was redundant across all seven proteomes (St2). These peptides are mainly derived from membrane proteins (PMA1, ion and molecule transporters), micronemes, and a hypothetical protein. These proteins corresponded to genes expressed in all three stages of the parasite (St2). Residue analysis at positions 4–6 indicated that three out of the five peptides contained amino acids that were positively associated with immunogenicity. All the peptides were predicted to undergo proteasomal cleavage (St2). Finally, alignment revealed that a 9-mer peptide (LYLLHSWTW) from an efflux transporter protein exhibited 77% identity with a human protein; therefore, it was excluded from the final list (St2). Regarding all peptides selected from Approach 1, including 9 AA sequences and 10 AA peptides (St1 and St2) a total of 22 peptides were found in the *T. gondii* proteomes (14 for the HLA-A*02 supertype, 4 for HLA-B*35, and 4 for HLA-A*24) (Supplementary Fig. 1, Sf1).

For Approach 2, beginning with the protein products of the parasite genes with high expression levels in human PBMCs infection, a total of 16 sequences were included in the analysis [47]. Starting from the reference genes in the RH strain, the corresponding sequences for the other strains were obtained from ToxoDB (ME49, GT1, VEG, ARI, MAS, VAND, and CATBr9) to perform epitope prediction. Based on these sequences, the prediction of 9-mer epitopes by “*Epitope Prediction*” library (IC < 50 nM) resulted in approximately 500 peptides per strain. Once evaluated in the 9-mer neural network, six to sixty peptides with the highest probabilities (*p* > 0.90) were identified. Upon visualization, these peptides exhibited some variability across the different positions (1–9); however, a higher frequency of leucine (L) at position 2 and valine (V) at position 9 was identified (Supplementary Fig. 2, Sf2). Regarding the 10-mer peptides analyzed using the same approach, approximately 300 epitopes per strain were predicted, of which 14–16 were classified by the network with the highest probabilities (*p* > 0.85). For these peptides, the sequence logos exhibited variability across all the positions. Nevertheless, the hydrophobic amino acids L, F, and V/W were predominantly observed at positions 2, 8, and 10, respectively (Sf3).

In the group of 9-mer peptides, three were found to be restricted to HLA-A*02 alleles and redundant across strains. With a lower cutoff (*p* > 0.60), three 9-mer peptides were identified for the HLA-B*35 alleles, which were redundant in at least four strains. Finally, for HLA-A*24, one redundant peptide was identified in seven strains and classified as positive by the network (IFWGILWFF) (St3). The seven identified 9-mer peptides corresponded to membrane proteins (ATPase-PMA1 and Toxofilin), rhoptry proteins, and a serine protease inhibitor protein. These proteins are encoded by genes expressed in all three stages of the parasite, according to ToxoDB. The peptides met most of the additional criteria for the in silico strategy, and none exhibited more than 70% identity with human sequences (St 3).

Regarding the 10-mer group in the second approach, four peptides were associated with the A*02 alleles and were redundant in at least six strains. For HLA-B*35, only one peptide was classified as positive by the neural network (FPLGSRFSPF) and was redundant in six strains. For HLA-A*24, two peptides were identified as redundant in at least four strains and were classified as positive by the network (St4). These peptides are also derived from membrane and surface proteins (PMA1 and SRS), an ROP kinase, and a PAN domain-containing protein that is involved in host cell interaction [61]. Regarding the final criteria, all peptides exhibited a positive proteasome cleavage score, and none showed identity with human sequences (St4). In summary, for Approach 2, 14 peptides of 9 and 10 amino acids were identified: seven for HLA-A*02, four for HLA-B35, and three for HLA-A24 (Sf1).

**Fig. 4:**
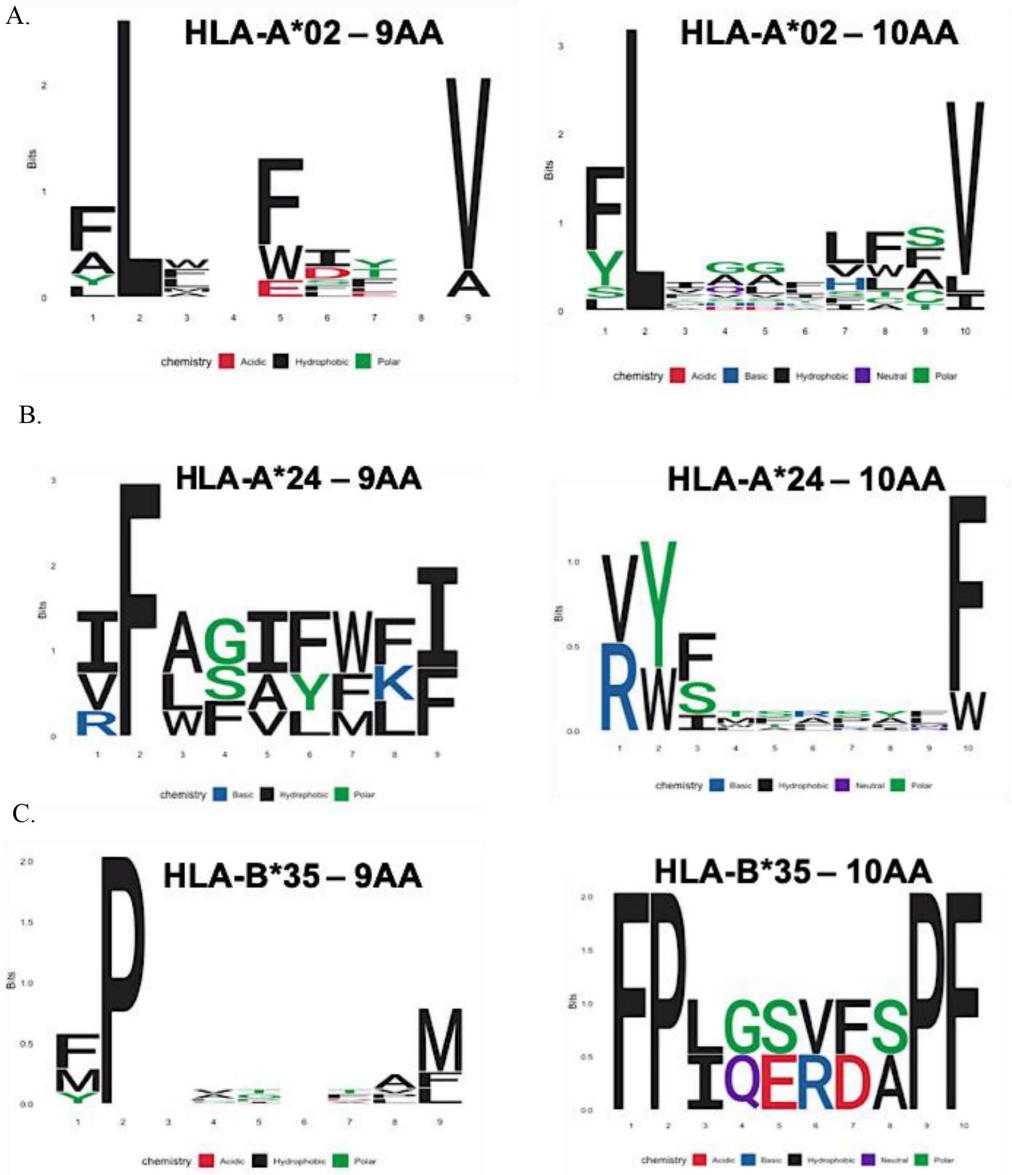
Sequence logos of selected peptides for HLA-A*02 (**A**), HLA-A*24 (**B**) and HLA-B*35 (**C**) supertypes are presented. These logos exhibit the chemical specificity of AA at positions 2 and the C-terminus, which are critical for interactions with the B and F pockets of each HLA allele

Overall, using the two different approaches, 36 peptides were identified from *T. gondii* proteins with affinity to three HLA-I frequent alleles, which have promising characteristics for experimental evaluation (Sf1). Additional sequence logos were performed for the selected peptides, in order to analyze if there was a differential pattern of amino acids depending on the supertype they were related, and interestingly, a chemical specificity of AAs was observed at the 2 and C-terminal positions important for interaction with the B and F pockets of each HLA-I (Fig. 4), the details of this analysis are described in the discussion section.

### Ex vivo analysis

#### Characterization of human samples

A total of 50 blood samples were analyzed from individuals who attended the CIBM (age range: 18–50 years, 64% [32/50] females). Informed consent and a survey were obtained from all participants. Among the evaluated samples, 40% (20/50) were seropositive for infection (anti-*Toxoplasma* IgG positive). Based on their serological status (anti-*Toxoplasma* IgM-negative) and the absence of any apparent symptoms or ocular lesions, seropositive individuals were classified as chronically asymptomatic (Supplementary Table 5, St5).

HLA-I typing using allele-specific PCRs revealed that 38% (19/50) of individuals were HLA-A*02 positive, displaying a characteristic band of approximately 734 bp (Sf4). For the HLA-A24 allele, a frequency of 68% (34/50) was observed with an expected band size of approximately 464 bp (Sf 5). Finally, 22% (11/50) of individuals were positive for the HLA-B*35 allele, presenting a band of 428 bp (Sf6.)

#### Evaluation of IFN-γ response

**Fig. 5:**
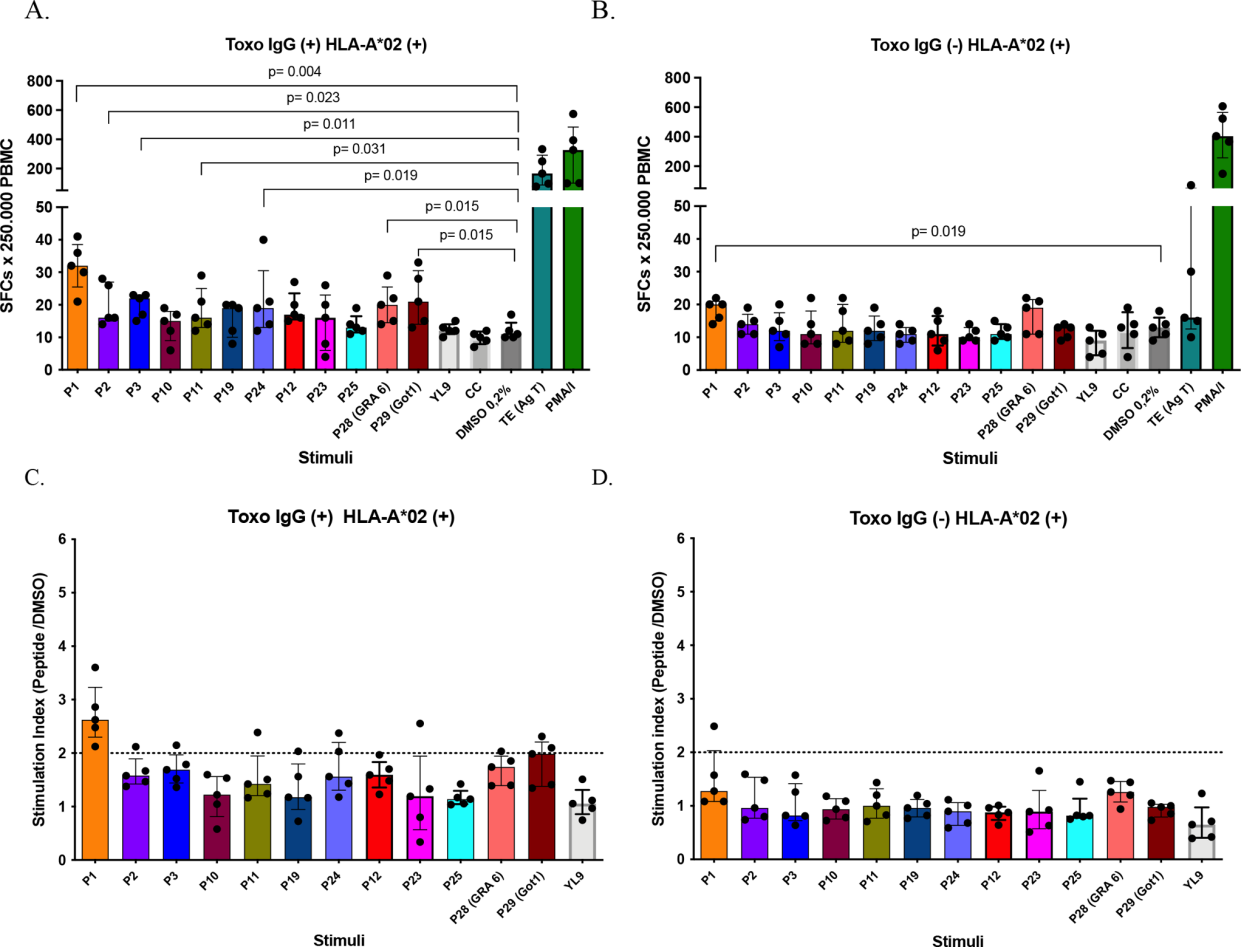
Number of IFN-γ producing cells detected by ELISpot in PBMC from HLA-A*02 + seropositive (IgG+, *n* = 5) (**A**), and seronegative (IgG-, *n* = 5) (**B**) individuals for *T. gondii*, after stimulation with 10 HLA-A*02-restricted peptides at 20 µg/ml and the respective controls. An irrelevant peptide (YL9) was used as a negative control, DMSO control (0.2%) and the no-stimulus cell control (CC). *T. gondii* total antigen (Ag T) and PMA/Ionomycin were used as positive controls. Mann-Whitney was performed to compare peptides vs. control (DMSO). Stimulation indices were obtained for the peptides in PBMC from seropositive (**C**) and seronegative (**D**) individuals. The index was calculated by dividing the number of spots generated by the stimulus (peptides) over those generated by the vehicle control (DMSO). Bars represent the median with the interquartile range

A total of 10 peptides with the best scoring characteristics for HLA-A*02, six for HLA-A*24, and eight for HLA-B*35 was selected for experimental validation (Supplementary Table 6, St6), from both strategies of selection. ELISpot assays performed to evaluate the IFN-γ response stimulated by peptides for the HLA-A*02 supertype in human PBMC, revealed that the peptide P1 (FLFAWITYV), derived from a zinc finger protein (Palmitoyl-transferase DHHC3) and selected through proteome-based approach 1, stimulated the highest number of IFN-γ-producing cells, showing a significant difference compared to the negative control (*p* = 0.004). This was followed by peptides P3 (*p* = 0.011), P24 (*p* = 0.019), P2 (*p* = 0.023), and P11 (*p* = 0.031) in the seropositive group (Fig. 5A). In the seronegative group, only peptide P1 showed a significant difference compared to the control (*p* = 0.019), although it was less significant (Fig. 5B). The Stimulation Index was also calculated to identify peptides considered immunogenic (SI > 2.0) [28]. Of the 10 peptides evaluated, only one (P1) induced stimulation index of 2 or higher in all seropositive individuals (Fig. 5C). In contrast, only one seronegative individual exhibited a stimulation index greater than 2 for this peptide (Fig. 5D), suggesting a specific immune response in the majority of seropositive individuals.

**Fig. 6:**
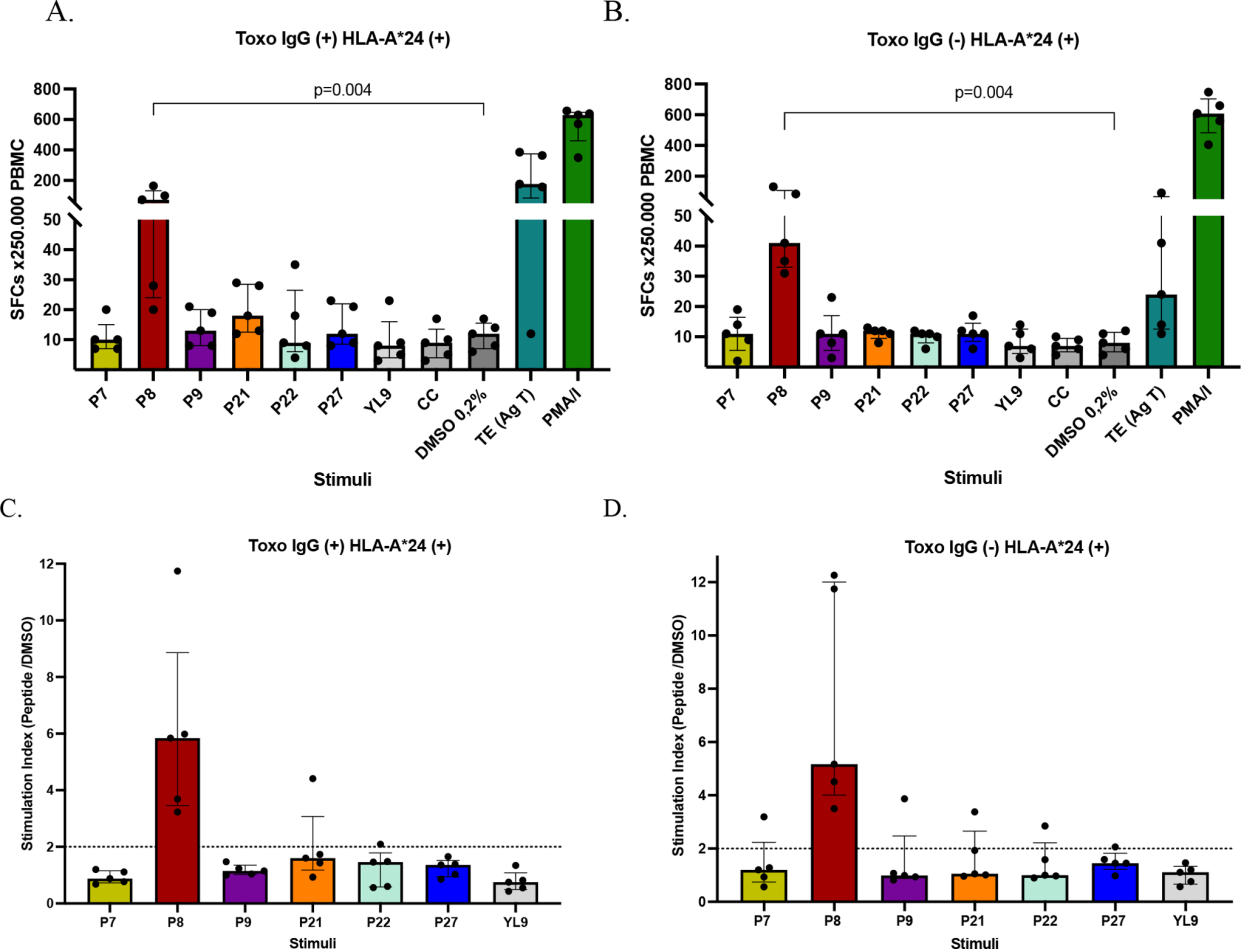
Number of IFN-γ producing cells detected by ELISpot in PBMC from HLA-A*24 + seropositive (IgG+, *n* = 5) (**A**), and seronegative (IgG-, *n* = 5) (**B**) individuals for *T. gondii*, after stimulation with 6 HLA-A*24-restricted peptides at 20 µg/ml and the respective controls. An irrelevant peptide (YL9) was used as a negative control, DMSO control (0.2%) and the no-stimulus cell control (CC). *T. gondii* total antigen (Ag T) and PMA/Ionomycin were used as positive controls for the assay. Mann-Whitney was performed to compare peptides vs. control (DMSO). Stimulation indices were also obtained for the peptides in PBMC from seropositive (**C**) and seronegative (**D**) individuals. Bars represent median with interquartile range

ELISpot assays were performed to evaluate the IFN-γ response stimulated by six peptides selected for the HLA-A*24 supertype in human PBMC that were positive for this allele. Among these peptides, only peptide P8 (VFAFAFFLI), derived from the potassium ion channel protein (PCIP) of *T. gondii*, induced significantly higher levels of IFN-γ-producing cells than the vehicle control (DMSO) in both seropositive (*p* = 0.004) and seronegative individuals (*p* = 0.004) (Fig. 6A and B). When the stimulation index was calculated, only P8 was found to be immunogenic, with values exceeding 2 in both *Toxoplasma* seronegative and seropositive individuals (Fig. 6C and D). This suggests that P8 activates an immune response involving innate immune cells in seronegative individuals and adaptive or memory immune cells in seropositive individuals.

**Fig. 7:**
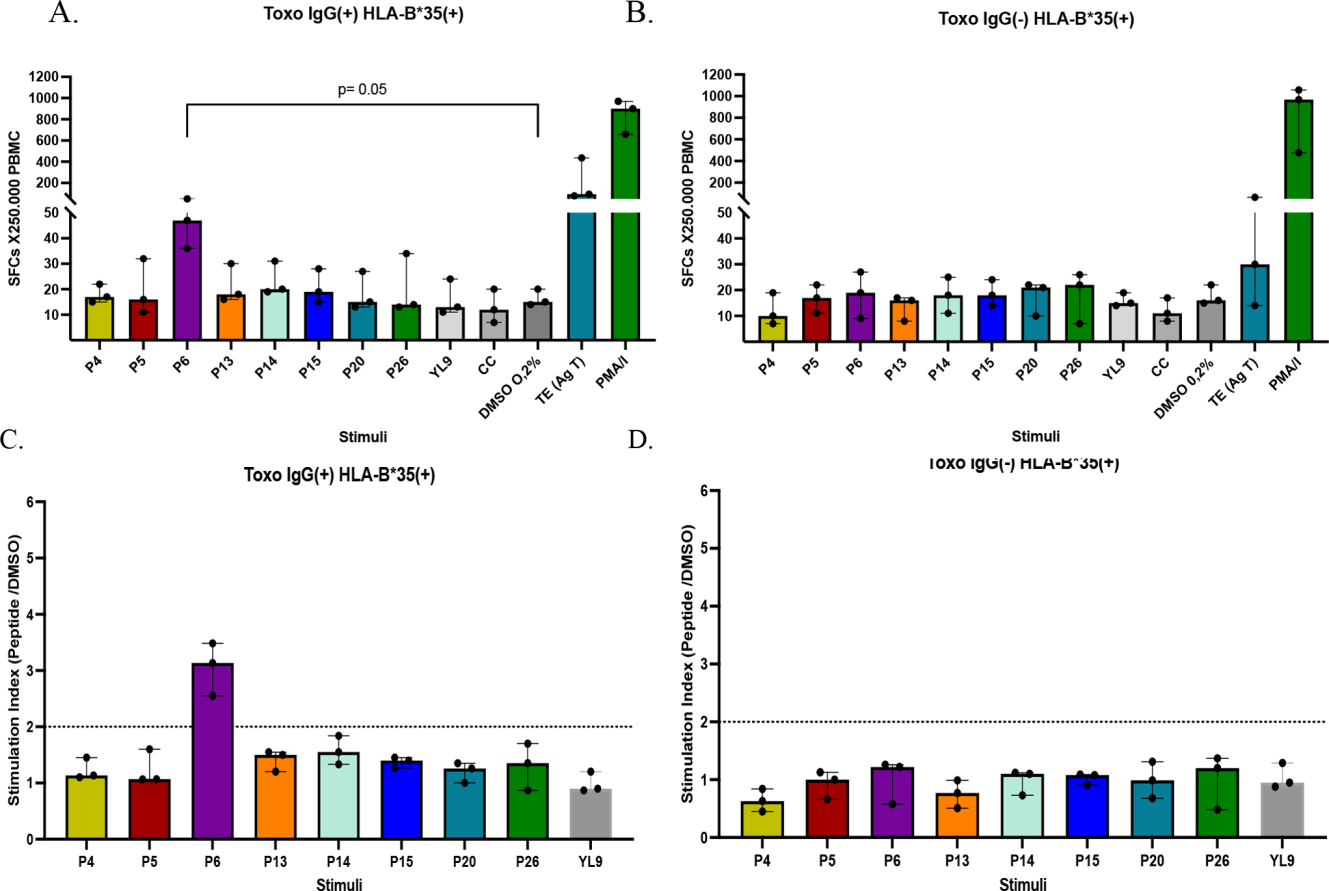
Number of IFN-γ producing cells detected by ELISpot in PBMC from HLA-B*35 + seropositive (IgG+, *n* = 3) (**A**), and seronegative (IgG-, *n* = 3) (**B**) individuals for *T. gondii*, after stimulation with 8 HLA-B*35-restricted peptides at 20 µg/ml and the respective controls. An irrelevant peptide (YL9) was used as a negative control, DMSO control (0.2%) and the no-stimulus cell control (CC). *T. gondii* total antigen (Ag T) and PMA/Ionomycin were used as positive controls for the assay. Mann-Whitney was performed to compare peptides vs. control (DMSO). Stimulation indices were also obtained for the peptides in PBMC from seropositive (**C**) and seronegative (**D**) individuals. Bars represent median with interquartile range

Regarding the results for the peptides restricted to HLA-B*35, eight peptides were evaluated in six individuals (three seropositive and three seronegative for *Toxoplasma*). This small group of individuals was studied because only 3 of the 50 individuals characterized were positive for both HLA-B*35 and IgG antibodies against *T. gondii*. Consequently, an equal number of seronegative individuals were included in this study. ELISpot assays revealed that peptide P6 (YPIAPSFAM), derived from a putative microneme protein of *T. gondii*, was the only peptide that significantly increased the number of IFN-γ-producing cells in the PBMC of individuals seropositive for toxoplasmosis (Fig. 7A), compared to the vehicle control (*p* = 0.05). In the seronegative group, none of the peptides elicited a significant response compared with the control (Fig. 7B). Among the stimulation indices for peptides with affinity for HLA-B*35, only peptide P6 was identified as immunogenic, displaying SI greater than 2 in seropositive individuals (Fig. 7C) and lower values in seronegative individuals (Fig. 7D).

**Fig. 8:**
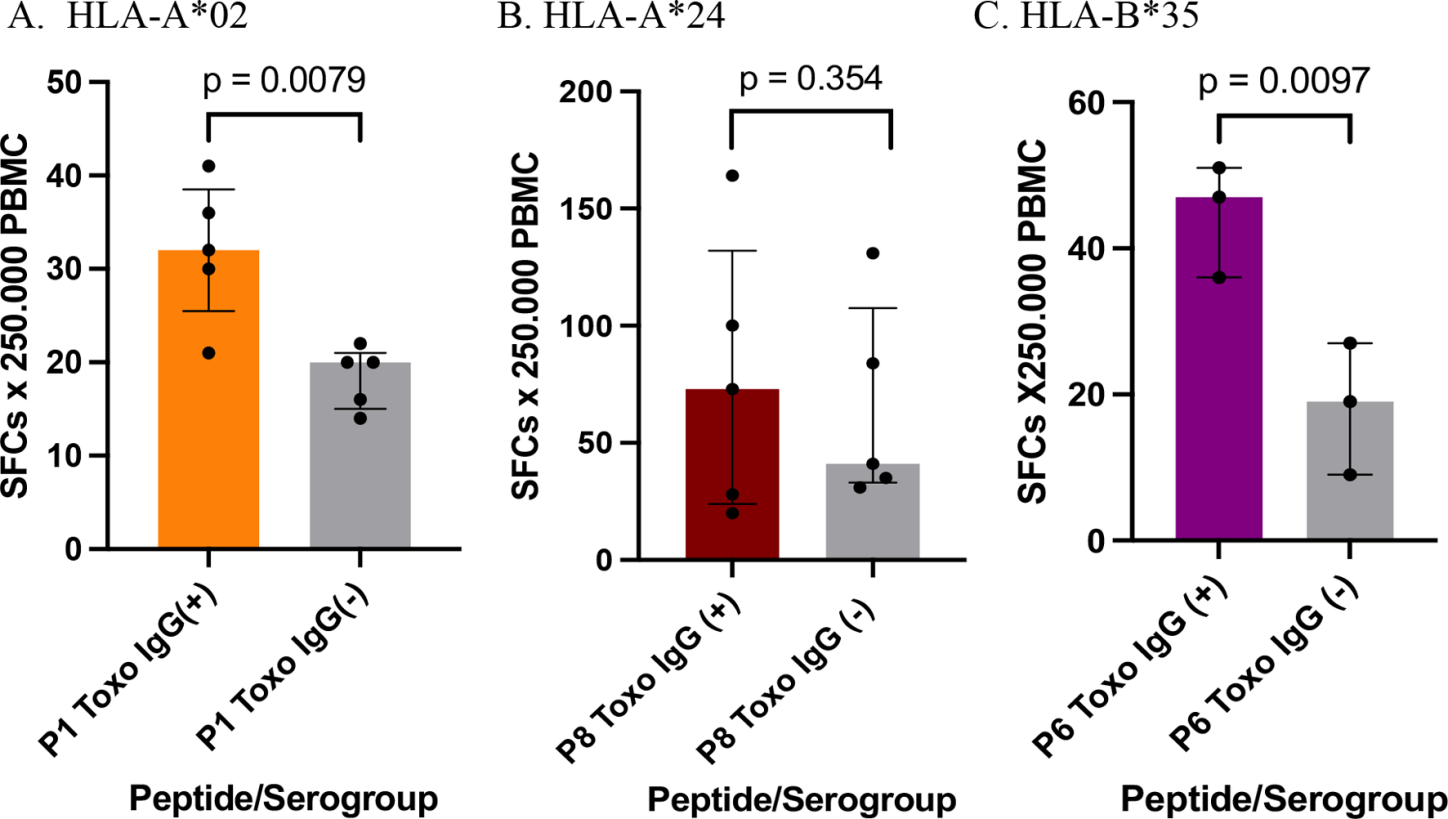
Comparison of IFN-γ producing PBMC between the seropositive (IgG+) and seronegative (IgG-) groups for *Toxoplasma gondii* was performed to assess the specificity of response stimulated by the most relevant peptides for each of the three HLA-I alleles, HLA-A*02 (**A**), HLA-A*24 (**B**) and HLA-B*35 (**C**). Mann-Whitney test was used to compare data. Bars represent the median values median with interquartile range

As a final analysis of ELISpot results, the data of the three most interesting peptides for each allele (P1-A*02, P8-A*24, and P6-B*35) were compared between the seropositive (IgG+) and seronegative (IgG-) individuals in order to assess the specificity of the IFN-γ response. We found that the IFN-γ response was significantly higher in seropositive individuals than in seronegative individuals for P1 (*p* = 0.007) and P6 (*p* = 0.009) (Fig. 8A and C). In contrast, for peptide P8, the response was high in both groups and similar between them (*p* = 0.354) (Fig. 8B). Therefore, P1 and P6 induced more specific IFN-γ responses.

### Evaluation of cytotoxic mechanisms (CD107a) of CD8 + T lymphocytes

Peptides P1, P8, and P6 were further characterized using flow cytometry to assess the activation of specific cytotoxic mechanisms in CD8 + T lymphocytes from both *T. gondii* seropositive and seronegative individuals. Flow cytometry was conducted in a small group of patients (*n* = 22). Degranulation was assessed using a lysosomal membrane-associated protein-1 (LAMP-1 or CD107a) marker, which is transported to the CD8 + T cell membrane upon release of cytotoxic granules [62]. The gating strategy and representative plots of the flow cytometry assay using PBMC from seropositive individuals are shown in Supplementary Fig. 7 (Sf7).

**Fig. 9:**
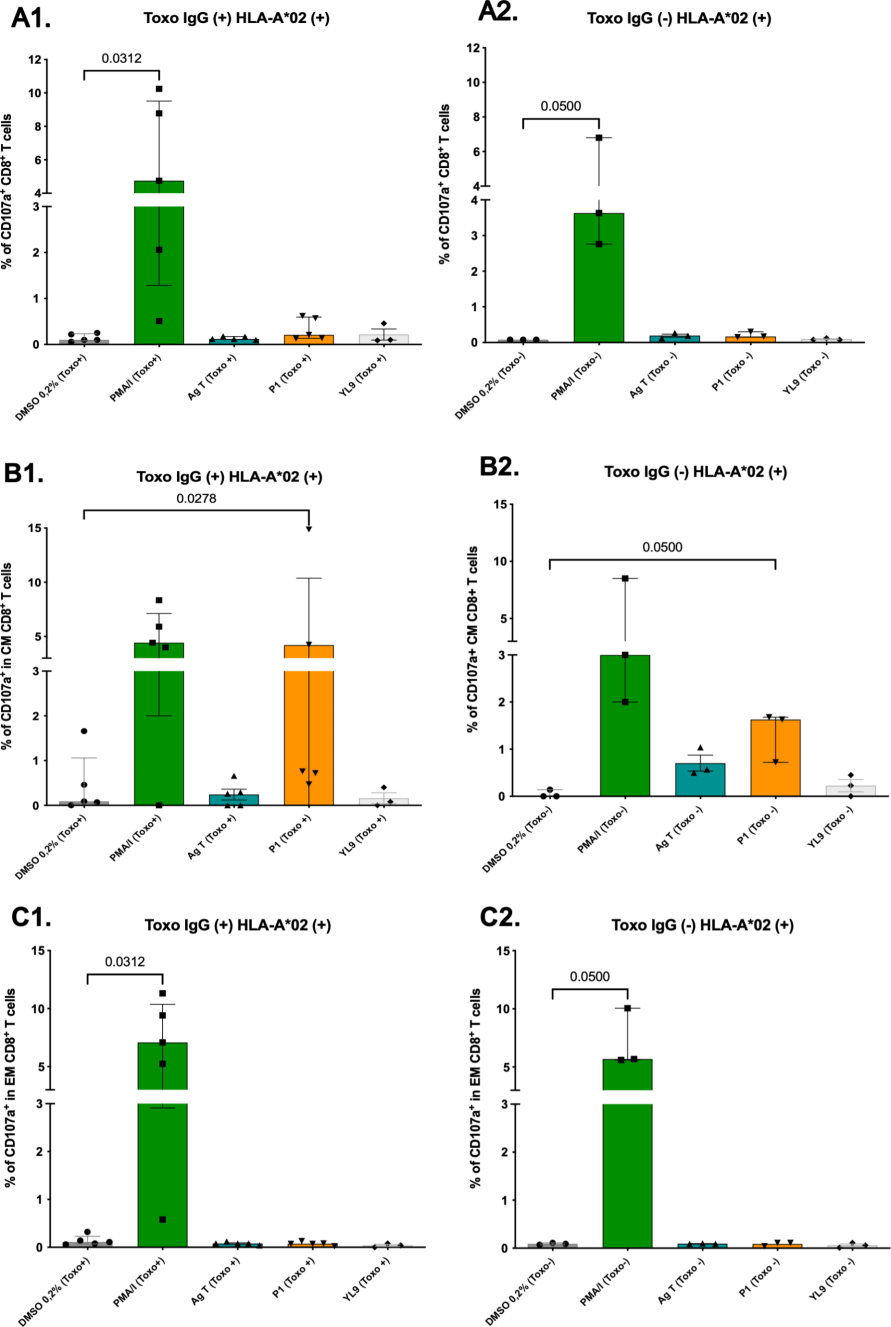
Populations of CD8 + T lymphocytes stimulated with T. gondii P1 peptide (FLFAWITYV) restricted to HLA-A*02 and the respective negative (DMSO and YL9) and positive (PMA/I) and Ag T controls for 6 h for detection of the degranulation marker CD107a (*n* = 8, 5 Toxo+ (IgG+) and 3 Toxo- (IgG-), HLA-A*02+). (**A**) Percentage of total CD8 + LT expressing CD107a in individuals (**A1**) seropositive and (**A2**) seronegative for Toxoplasma are presented. (**B**) % of central memory CD8+ (CM) and (**C**) % of effector memory CD8 + cells (EM). Bars represent median with interquartile range. Mann-Whitney test was performed to compare data of stimulus Vs. control (DMSO)

In the HLA-A02 + group and *Toxplasma* seropositive individuals, peptide P1 (FLFAWITYV) stimulated CD107a expression in a small percentage of total CD8 + T cells (median [Me] = 0.21%, Interquartile Range [IQR] = 0.13–0.59%) (Fig. 9A). However, degranulation was significantly higher in the central memory CD8 + T cell population (CM, CD45RAneg/CCR7pos) (Me = 4.21%, IQR = 0.59–9.55%) compared to the control (DMSO) (*p* = 0.0278) (Fig. 9B1). In addition, CD107a expression was observed in a small percentage of effector memory CD8 + T cells (EM, CD45RAneg/CCR7neg) (Me = 0.07%, IQR = 0.04–0.10%) (Fig. 9C1). In the seronegative group, peptide P1 (FLFAWITYV) induced modest CD107a expression in CD8 + and CD8 + EM T cells (Me = 0.17% and 0.11%, respectively) (Fig. 9A2 and C2). Higher degranulation was observed in the CM CD8 + T cell population (Me = 1.63%, IQR = 0.72–1.68%), although no significantly statistical differences were found compared to the control (*p* = 0.05) (Fig. 9B2).

**Fig. 10:**
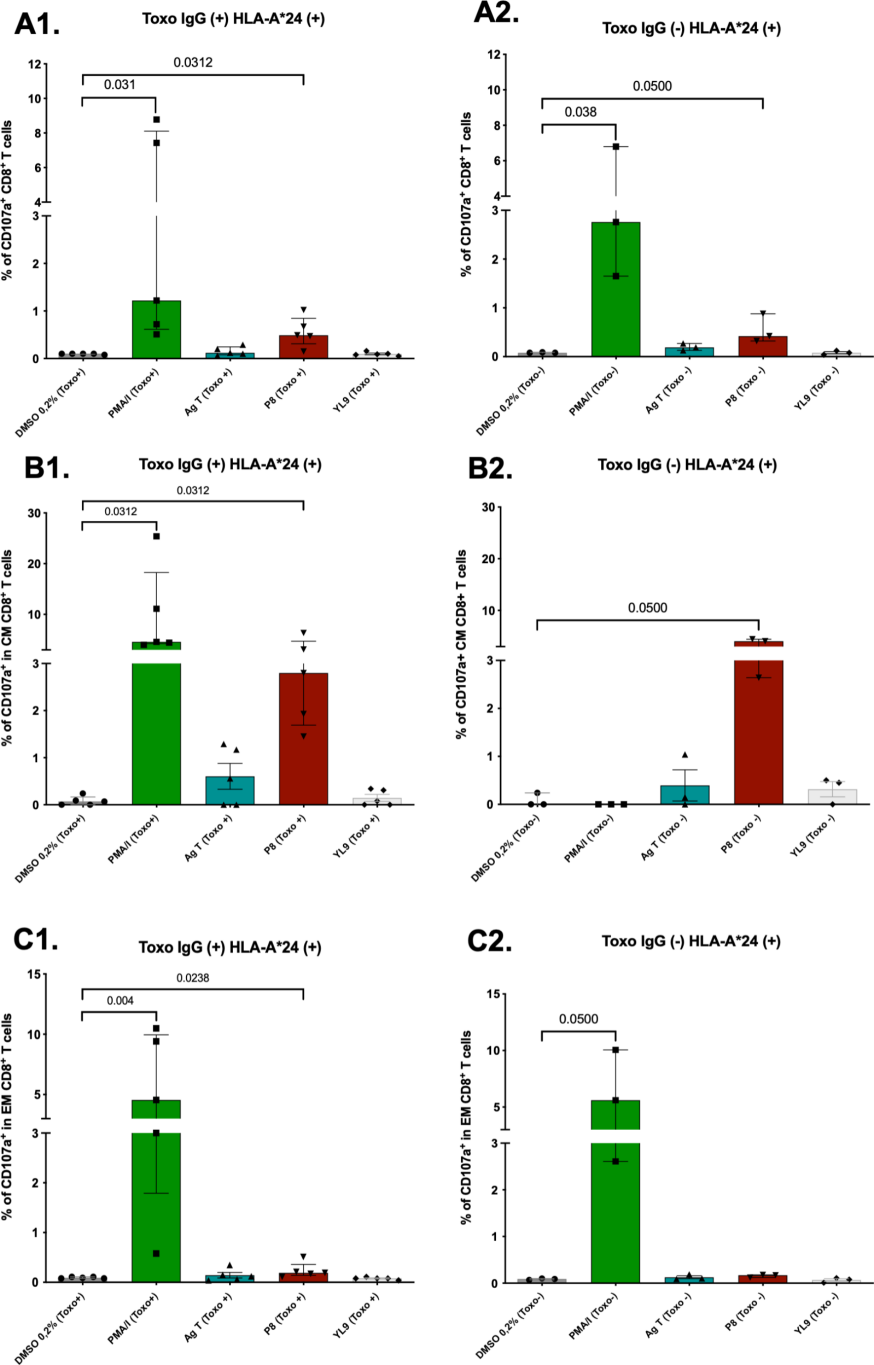
Populations of CD8 + T lymphocytes stimulated with the T. gondii P8 peptide (VFAFAFFLI) restricted to HLA-A*24 and the respective negative (DMSO and YL9) and positive (PMA/I) and Ag T controls for detection of the CD107a marker (*n* = 8, 5 Toxo+ (IgG+) and 3 Toxo- (IgG-), positive for HLA-A*24). (**A**) % of total CD8 + cells expressing CD107a in individuals (**A1**) seropositive and (**A2**) seronegative for Toxoplasma. (**B**) % of central memory (CM) CD8 + cells and (**C**) % of effector memory (EM) CD8 + cells. Bars represent median with interquartile range. Mann-Whitney test was performed to compare stimulus Vs. control (DMSO)

Flow cytometry assays of HLA-A*24 + individuals stimulated with P8 (VFAFAFFLI) demonstrated cytotoxic response activation in some cell populations. In the seropositive group, peptide P8 induced CD107a expression in a modest percentage of total CD8 + T cells (Me = 0.49%, IQR = 0.31–0.84%), showing significant differences compared to the negative control (*p* = 0.031) (Fig. 10A1). In the CD8 + CM T cell population, degranulation marker expression was significantly higher (Me = 2.80%, IQR = 1.69–4.72%) than in the vehicle control (Me = 0.07%, IQR = 0–0.16%) (*p* = 0.031) (Fig. 10B1). In the CD8 + EM T cell population, although the degranulation response was lower (Me = 0.19%, IQR = 0.14–0.36%), significant differences were observed compared to the negative control (*p* = 0.023) (Fig. 10C1). In the HLA-A*24 + seronegative group, P8 induced CD107a expression in a small percentage of total CD8 + T cells (Me = 0.42%, IQR = 0.32–0.84%), without differences against the control (*p* = 0.05) (Fig. 10A2). For CD8 + CM T cells, the response was more pronounced (Me = 4.04%, IQR = 2.64–4.44%), with significant differences compared to the 0.2% DMSO control (*p* = 0.05) (Fig. 10B2). In CD8 + EM T cells, CD107a expression following peptide stimulation was detected in a lower percentage of cells (Me = 0.17%, IQR = 0.12–0.18%) (Fig. 10C2).

**Fig. 11:**
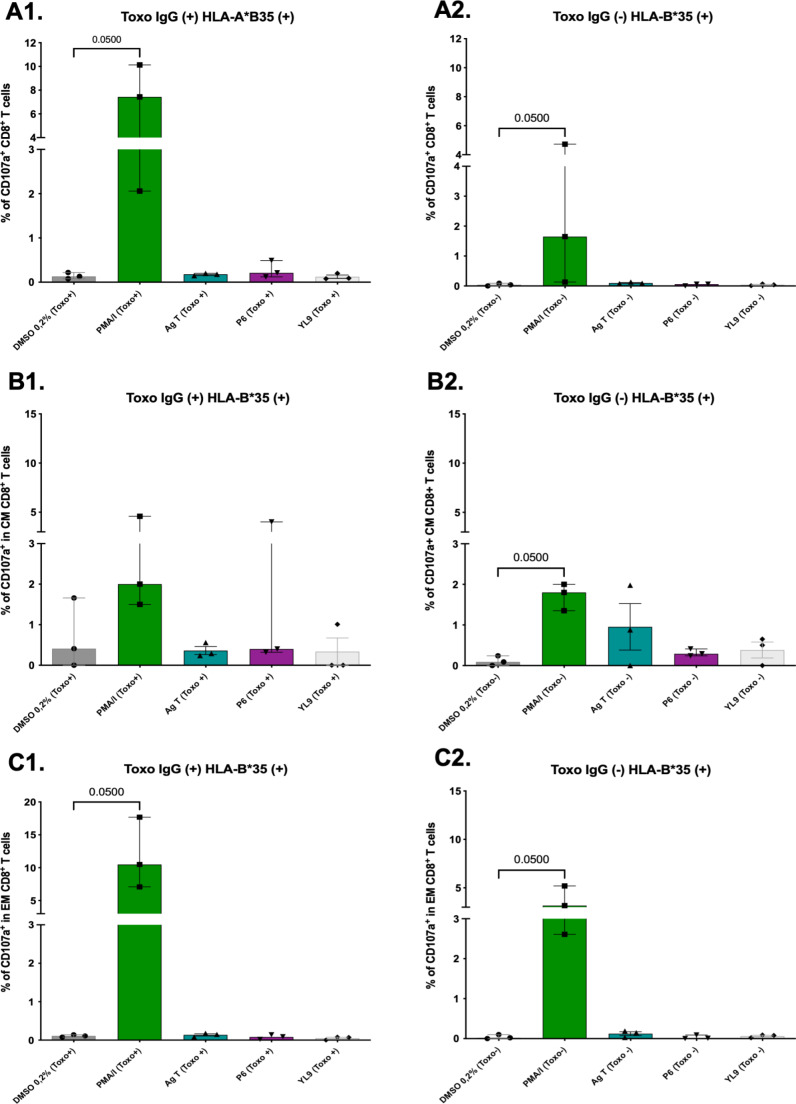
Populations of CD8 + T lymphocytes stimulated with the HLA-B*35-related T. gondii P6 peptide (YPIAPSFAM) and the respective negative (DMSO and YL9) and positive (PMA/I) and Ag T controls for detection of the CD107a marker (*n* = 6, 3 Toxo+ (IgG+) and 3 Toxo- (IgG-), positive for HLA-B*35). (**A**) % of total CD8 + cells expressing CD107a in individuals (**A1**) seropositive and (**A2**) seronegative for Toxoplasma. (**B**) % of central memory (CM) CD8 + cells and (**C**) % of effector memory (EM) CD8 + cells. Bars represent median with interquartile range. Mann-Whitney test was performed to compare stimulus Vs. control (DMSO)

In the HLA-B*35 + group (*n* = 6), peptide P6 (YPIAPSFAM) was evaluated. Among the three *Toxoplasma*-seropositive individuals, P6 induced modest CD107a expression in a small subset of total CD8 + T cells (Me = 0.21%, IQR = 0.12–0.49%) (Fig. 11A1). Although a slightly higher proportion of CD107a-positive cells was observed in the CD8 + CM T cell population (Me = 0.40%, IQR = 0.32–4.0%), statistical significance was not reached due to data variability (Fig. 11B1). In the CD8 + EM T cell population, P6 induced a minimal percentage of cells (Me = 0.09%, IQR = 0.02–0.14%) (Fig. 11C1). In the seronegative group, peptide P6 induced minimal CD107a expression in both total CD8 + and EM T cells (Me = 0.06% and 0.01%, respectively) (Fig. 11A2 and C2). A slightly higher percentage of CD8 + CM T cells exhibited CD107a expression (Me = 0.29%, IQR = 0.24–0.41%) (Fig. 11B2).

To conclude the flow cytometry data analysis, we compared the cytotoxic responses of CD8 + CM cells from the seronegative and seropositive (chronic-asymptomatic) groups for toxoplasmosis to evaluate the specificity of responses stimulated by *T. gondii* peptides. A higher percentage of CD8 + central memory cells expressing CD107a was generally observed in the seropositive group; however, no significant differences were detected between the two groups for any of the three evaluated *T. gondii* peptides (Table 2).

**Table 2:** Statistical comparison of responses observed between seropositive (Toxo-IgG+) and seronegative (Toxo-IgG-) individuals in flow cytometry assays, specifically comparing the median of cell percentages of CD8 + central memory (CM) cells expressing CD107a after peptide stimulation

Assay	Peptide	Allele HLA-I	Serogroup		Mann-whitney test *P*-value
			Toxo-IgG (+) Me (IQR)	Toxo-IgG (-) Me (IQR)	
Flow Cytometry:	P1	HLA-A*02+	**4.2** (0.59–9.55)	1.3 (0.72–1.68)	0.232
CD8^+^ CM %CD107a+	P8	HLA-A*24+	**3.7** (2.64–4.44)	3.1 (1.69–4.72)	0.354
(*n* = 22)	P6	HLA-B*35+	**0.40** (0.2–4,01)	0.29 (0.24–0.41)	0.179

## Discussion

The search and identification of immunogenic peptide epitopes has emerged as a promising strategy for developing a multi-epitope vaccine against toxoplasmosis [57, 63–65]. In this context, the application of bioinformatics tools in immunology, combined with reverse vaccinology, has significantly impacted the discovery of new candidates owing to its rapid and promising outcomes [66–69]. These approaches facilitate comprehensive scanning of potential pathogen antigens through molecular modeling, enabling the identification of interactions with host receptors and major histocompatibility complexes [70]. Additionally, Artificial Intelligence (AI) methods, such as *Deep Learning* and *Artificial Neural Network* architectures, have the potential to automate epitope identification by analyzing large datasets, thus accelerating the discovery of more effective vaccine candidates [71].

Recognizing the potential of in silico analysis, the first part of this study involved developing Artificial Neural Network (ANN) architectures and utilizing various bioinformatics tools to predict and select immunogenic peptides from *T. gondii*. This was accomplished either by analyzing the complete proteome of different strains of the parasite or by focusing on protein products from overexpressed genes in the protozoan during infection of human PBMC. Peptides with the highest probability of interacting with relevant HLA-I complexes that met the majority of the criteria in the in silico strategy were selected.

Interestingly, the 9-mer peptides with the highest predicted probabilities by ANNs across the seven parasite strains showed a consistent pattern of hydrophobic and aliphatic amino acids, such as leucine (L) and valine (V), at positions 2 and 9, respectively (Fig. 3A). Similarly, for 10-mer peptides, a pattern of hydrophobic, aliphatic, and aromatic residues (F and L) was observed at positions 1 and 2, with V and L at the C-terminal position (Fig. 3B). These findings are significant because this pattern of hydrophobic amino acids at these positions correspond to anchoring sites within HLA-I binding pockets [60]. It has also been reported that the B and F pockets of HLA-I, which interact with residues at these positions, exhibit chemical specificity for aliphatic, aromatic, and predominantly hydrophobic amino acids [72].

Additionally, we observed minor variations in the chemical specificities of the residues based on the HLA supertype (Fig. 4). For HLA-A*02, leucine (L) and valine (V) were predominantly found at these positions (Fig. 4A), in agreement with Sette et al. [72], where pocket B interacts with small aliphatic residues (such as L) and pocket F is specific for aliphatic and small hydrophobic residues (such as V) [72]. For the HLA-A*24 supertype, residues F/Y/W were identified at position 2 and I/F/W at the C-terminal position (Fig. 4B), which aligns with the chemical specificity of pocket B for aromatic residues (such as F and W), and with the broader specificity of pocket F, which interacts with aromatic, aliphatic, and hydrophobic residues [72]. Finally, for HLA-B*35, all the selected peptides contained proline (P) at position 2 and phenylalanine (F) at the C-terminal position (Fig. 4C). Sette et al., similarly reported specificity for proline in pocket B of the B*35 supertype, and affinity for aromatic, aliphatic, and hydrophobic residues in pocket F [72].

Beyond identifying the properties of peptides for potential HLA-I interactions, we observed that positions 4–6 in most of the epitopes with the highest predicted probabilities contained at least one of the following residues: F, I, W, or A. These residues have previously been linked to immunogenicity [28]. It has been described that, aromatic side chains, such as phenylalanine (F) and others (I, W, A), are positively associated with immunogenicity [49]. Conversely, small residues such as serine (S), K, M, and Q, are negatively associated with this characteristic [49]. Notably, it has been reported that amino acids at positions 4–6 are critical for interaction with T-cell receptor (TCR) residues and are therefore essential for activating the immunogenic response [49]. Functional evidence of this interaction may be linked to our experimental results, particularly by the production of IFN-γ, which is likely produced by CD8 + T cells from seropositive individuals that recognize *T. gondii* peptides.

Additionally, a key aspect of our in silico strategy was the review and characterization of the proteins from which the predicted peptides were derived. These proteins are located in the membrane and secretory organelles, such as rhoptries and micronemes, as well as in the cytosol, endoplasmic reticulum, nucleus, and mitochondria, which are associated with metabolic and catalytic functions, as previously reported by Cardona et al. [28]. and McMurtrey et al. [23]. This suggests that not only surface or secreted proteins are crucial for inducing an immune response in humans. This is particularly significant, as most of the epitopes evaluated for *T. gondii* to date have been derived from surface antigens and secretory proteins, such as SAG, GRA, ROP, MIC proteins, and more recently, ROM and MAG antigens [73]. Therefore, this study broadens the scope of potential proteins and peptides that may naturally activate cellular immune responses against *T. gondii.*

Moreover, we found that the genes encoding the proteins from which the peptides were derived were expressed across all three parasite stages that affect humans— oocysts, bradyzoites, and tachyzoites —and that the peptides were conserved among the different *T. gondii* strains analyzed (according to ToxoDB). These features make the peptides promising candidates for future vaccine development, as they may induce an immune response against infections at any stage or strain (typical or atypical) of the parasite [74]. Other studies have focused on identifying conserved regions as potential vaccine candidates. A recent in silico study aimed at designing a multi-epitope subunit vaccine for visceral leishmaniasis selected conserved B and T cell epitopes from four different *Leishmania* strains derived from four distinct antigenic proteins [75], and demonstrated that the vaccine triggered a pro-inflammatory response, including the proliferation of activated T and B lymphocytes, suggesting a promising vaccine construct.

After characterizing the proteins from which the peptides were derived, additional conditions were analyzed in the selection strategy. One of these conditions addresses a key feature of immunogenic peptides processed via the MHC-I pathway: a positive prediction for proteasomal cleavage and TAP transport. As described in the literature, in the conventional MHC class I antigen processing pathway, proteins are degraded by proteasomes into fragments of approximately 3–18 amino acids, only a subset of which reaches the transporter associated with antigen processing (TAP) located in the endoplasmic reticulum (ER) membrane. In the ER, these peptides are loaded onto MHC I molecules before being transported to the cell surface for presentation to T cells [25, 76]. A previous study found that approximately 15% of all peptides generated from a protein were successfully transported to the ER, with approximately 2.5% binding to an MHC molecule. Ultimately, approximately 50% of the peptides present on the cell surface are recognized by T cell receptors [78]. Thus, efficient prediction is crucial for identifying peptides that can effectively activate the T lymphocytes. At this step of the analysis, the complete sequences of the *T. gondii* proteins from which the peptides were derived were loaded into IEDB analysis resources, utilizing the combined antigen-processing predictor [77]. In this web server, proteasomal cleavage was predicted, where the score indicated the likelihood that the peptides contained the necessary residues for cleavage at the C-terminal end. Additionally, TAP transport prediction assessed the ability of the peptide to be generated with its extended N-terminal precursor [78]. Interestingly, all peptides previously selected by neural networks showed a positive score for proteasomal cleavage and TAP transport (total score > 0.5). Therefore, no peptides were excluded at this stage of the analysis.

As the final criterion in the in silico analysis, peptide alignment with human protein sequences was performed to eliminate those with identities greater than 70%, to avoid cross-reactivity and potential autoimmune responses. This threshold was chosen based on recent studies on epitope-based vaccine development targeting other infectious diseases. For example, in Chagas disease, *T. cruzi* epitopes for T and B cells were identified for inclusion in a multi-epitope vaccine, ultimately retaining 18 peptide sequences with identities below 70% to any human protein or microbiome-associated protein [79]. In our study, some peptides predicted by the neural network were excluded, including one derived from the RON3 protein (FLLDFLLYV) and another from an efflux transporter protein (LYLLHSWTW), as they showed 77% or higher identity with human proteins.

Regarding the experimental phase of the study, characterization of eligible individuals revealed that 38% were positive for the HLA-A*02 allele, 68% were positive for the HLA-A*24 allele, and 22% were positive for the HLA-B*35 allele. These frequencies are generally consistent with previous reports for A*02 (23–50%) and B*35 (16–23%), but the A*24 frequency was higher than those reported in studies from Colombia, South America, and other regions (20–36,5%) [28, 34, 36–39, 80–83]. This suggests that the HLA-A*24 supertype is more prevalent in our study population [54].

Following the identification of peptides and selection of individuals with relevant HLA-I alleles, peripheral blood mononuclear cells (PBMC) from individuals previously infected with *T. gondii* were used as a model to assess the immune response stimulated by the peptides. As reported in the literature, PBMC immune responses to *T. gondii*-derived peptides are predominantly characterized by elevated IFN-γ levels [84]. Specifically, in order to find immunogenic peptide epitopes, we decided to test our selected peptides with people with chronic asymptomatic infection because they have successful control of the infection and did not show pathological consequences (e.g., retinochoroiditis). Therefore, ELISpot and flow cytometry assays were developed with PBMCs from these individuals, in order to evaluate peptide-stimulated cytokine production and cytotoxic responses. The 9mer and 10mer aminoacids peptides were evaluated in ex vivo assays, but only those with 9 residues stimulated a relevant IFN and cytotoxic response.

Regarding the results of the ELISpot assay revealed that peptide P1 (FLFAWITYV), related to HLA-A*02, derived from the palmitoyl-transferase DHHC3 protein of *T. gondii*, induced an average of approximately 40 spot-forming cells (SFCs) per 250,000 PBMCs (Fig. 5A). When extrapolated to 1 × 10^6^ of PBMC per well, as reported in previous studies [28, 64], this corresponds to roughly 160 SFCs. Similarly, peptide P8 (VFAFAFFLI), derived from a potassium ion channel protein and restricted to HLA-A*24, generated a stronger response (Fig. 6A) with an extrapolated value of 400–600 SFCs per 1 × 10^6^ de PBMC. Peptide P6 (YPIAPSFAM), derived from a putative microneme protein restricted to HLA-B*35, showed an extrapolated value of approximately 200 SFCs per/1 × 10^6^ PBMC (Fig. 7A). These values are comparable to those obtained in prior IFN-γ ELISpot assays for immunogenic *T. gondii* peptides restricted to HLA-A*11:01 tested in PBMC from seropositive individuals [64]. This study evaluated five peptides from membrane and secretory proteins, including SAG1_224 − 232_(KSFKDILPK), SAG2C_13 − 21_(STFWPCLLR), GRA5_89 − 98_(AVVSLLRLLK), SRS52A_250 − 258_ (SSAYVFSVK), and GRA6_164 − 172_ (AMLTAFFLR), which stimulated approximately 100–200 SFCs per 1 × 10^6^ de PBMC [64]. This indicates that our peptides, selected through the rational strategy and derived from cytosolic or metabolism-associated proteins, elicit immune responses comparable to those generated by secretory or surface proteins of the parasite, which are recognized as immunogenic.

It is important to emphasize that the selection of peptides restricted to HLA-I was intended to activate the cellular response primarily mediated by CD8 + T lymphocytes (TL), which have been recognized as crucial for protection against *T. gondii* [21]. CD8 + TL mediate their effector functions through the production of cytokines such as IFN-γ and tumor necrosis factor-alpha (TNF-α), and/or through cytolytic mechanisms, where cytotoxic T lymphocytes (CTL) are the protagonists. These responses are crucial for maintaining control not only against *T. gondii* but also against a range of intracellular infections [85]. The CTL response is the key effector arm of the immune system for protective immunity to control intracellular infections and is elicited by small linear epitopes from processed antigens [86].

When CD8 + TL were stimulated, the predominant cells involved in the cytotoxic response, as evaluated by flow cytometry, were central memory (CM) CD8 + T cells, which responded more robustly to stimulation with P1, P6, and P8 peptides in most of the individuals assessed. Although the magnitude of this activation was relatively low (< 5%) in terms of the percentage of CD8 + T cells positive for the degranulation marker CD107a (Figs. 9 and 10, and 11), there were significant different when compared with the negative control. Additionally, the observed percentages of CD107a + cells were comparable to those previously reported for human PBMC stimulated with cytomegalovirus peptides (~ 0.42%) [58].

We found a higher proportion of CD107a-positive CM CD8 + T cells responding to peptide stimulation than effector memory (EM) T cells, which is consistent with a recent study that evaluated *Leishmania braziliensis* epitopes in central and effector memory CD4 + and CD8 + T cells from individuals with cutaneous leishmaniasis and healthy controls. The study reported significantly higher frequencies of CM CD8 + cells in affected individuals following peptide stimulation, indicating that these cells act as reservoirs of antigen-specific cells [87]. Additionally, it has been noted that CM T cells have greater proliferative capacity compared to EM T cells after antigenic stimulation, especially when previously exposed to the same antigenic source [88]. Therefore, our peptides could induce a peptide-specific cellular response and proliferation of CD8 + CM T cells.

On the other hand, peptide P8 induced response in PBMC from both, chronically asymptomatic and seronegative individuals with no statistically significant differences between the groups (Fig. 8). To explain the promiscuous behavior of this peptide, we hypothesized that P8 may activate not only memory T lymphocytes in seropositive individuals, but also innate immune cells in seronegative individuals. It is plausible that the peptide interacts with other innate immune receptors [89], potentially triggering the expression of transcription factors and the transcription of cytolytic effector genes, leading to the production of proinflammatory cytokines and/or the release of cytotoxic granules [90]. The analysis of these innate immune cells in seronegative individuals stimulated with P8 is a perspective of this study.

The final aspect of the analysis focused on understanding how the proteins from which peptides were derived entered the antigen MHC-I processing pathway. P6, originating from an MIC-type protein, is likely to be secreted into the host cell by secretory organelles, micronemes. The protein probably follows the conventional MHC-I processing pathway, consistent with the literature and most reports on *T. gondii* [25, 73]. In contrast, P1 and P8, which are derived from the cytoplasmic proteins of the parasite, it is hypothesized to have been processed via alternative mechanisms, such as autophagy. Autophagy is an intrinsic cellular recycling system [76], a catabolic pathway generally used to degrade cytoplasmic material, and is capable of mediating pathogen destruction [91]. It can be stimulated by both the innate and adaptive immune responses [92]. It has been proposed that autophagy can intersect with the MHC-I presentation pathway to alert CD8 + T cells to intracellular infections [76]. Based on this hypothesis, autophagy likely plays a dual role, not only in facilitating intracellular pathogen destruction but also in enhancing immune surveillance by presenting pathogen-derived peptides, as seen with *T. gondii* in this case.

Considering these results, P1, P6, and P8 peptides from *T. gondii* could be part of a multi-epitope vaccine candidate that can activate memory and innate immune responses. The literature reports that multi-epitope vaccines more accurately mimic antigen processing and presentation during a natural infection, and thus induce more efficient protective immunity than whole-protein vaccines [19]. Due to the complexity of the parasite life cycle and the variability of parasite antigens, the development of multi-epitope vaccines against *T. gondii* is an attractive alternative to previous vaccine approaches [17].

In summary, this study offers valuable contributions to the discovery, identification, and evaluation of *T. gondii* immunogenic peptides as potential components of future multi-epitope vaccines against human toxoplasmosis. This study integrated the application of in silico strategies with ex vivo methodologies, employing human PBMC models and various immunological techniques. However, additional methodological approaches, such as analyzing granzyme B and perforin as part of cytotoxic activity and other Th1 cytokines, studying larger populations, and utilizing animal models, are necessary to validate the proposed peptide candidates further. Furthermore, this combined in silico and ex vivo approach could be applied to other pathogenic microorganisms in order to find peptide sequences that could trigger an immune response.

## Electronic supplementary material

Below is the link to the electronic supplementary material.

Supplementary Material 1

Supplementary Material 2

## Data Availability

No datasets were generated or analysed during the current study.
