# Supplementary material for: In silico identification and ex vivo evaluation of Toxoplasma gondii peptides restricted to HLA-A*02, HLA-A*24 and HLA-B*35 alleles in human PBMC from a Colombian population
Source: Med Microbiol Immunol. 2024 Dec 31;214(1):5. doi: 10.1007/s00430-024-00815-x (PMC11688256; doi:10.1007/s00430-024-00815-x)
Supplement: Supplementary file 1 — Supplementary Material 1 [file 430_2024_815_MOESM1_ESM.docx]

*In silico* identification and *ex vivo* evaluation of *Toxoplasma gondii* peptides restricted to HLA-A*02, HLA-A*24 and HLA-B*35 alleles in human PBMC from a Colombian population

Journal name: Medical Microbiology and Immunology

Mónica Vargas-Montes https://orcid.org/0000-0003-2418-3596^1^, María Camila Valencia-Jaramillo^1^, Juan David Valencia-Hernández https://orcid.org/0000-0002-9189-5684^1^, Jorge Enrique Gómez-Marín https://orcid.org/0000-0001-6472-33291 Ailan Farid Arenas https://orcid.org/0000-0002-0331-5556^1^, Néstor Cardona https://orcid.org/0000-0002-6967-165X^1,2*^

^1^ Grupo de Estudio en Parasitología Molecular (GEPAMOL), Faculty of Health Sciences, Centro de Investigaciones Biomédicas, Universidad del Quindío, Quindío, Armenia, Colombia.

^2^ Faculty of Dentistry, Universidad Antonio Nariño, Quindio, Armenia, Colombia.

* Corresponding author:

*E-mail address*: nestorcardonape@uan.edu.co, nicardona@uniquindio.edu.co

Supplementary tables (St) and figures (Sf)

St. 1. Peptides of 9 AA predicted by the neural network in Approach 1. The probability for each peptide, some characteristics of the proteins from which they were derived according to ToxoDB, and the most important criteria of *in silico* analysis are presented. All peptides were conserved among the seven strains analyzed.

| No. | Peptide | Probability  (X̅ +/- SD) | Alleles-affinity | Code Toxo DB and Protein | Expression/Stage^A^ | Subcellular Localization ^B^ | TCR- immunogenicity  (presence of AA) ^C^ | Cleavage prediction: proteasome/TAP (score > 0,5) ^D^ | BLASTp ^D^  Identity >70% with humans |
| --- | --- | --- | --- | --- | --- | --- | --- | --- | --- |
| 1 | FLFAWITYV | 0.999  +/- 6.38E-05 | HLA-A*02 | TGME49_217870 DHHC3 Palmitoyl transferase | 3 stages  (Higher in Oocyst) | Membrane  Golgi A. | Yes | Positive  (score: 1.10) | No |
| 2 | FLFSFFFFV | 0.998  +/- 8.05E-05 | HLA-A*02 | TGME49_213100 Hypothetical protein | 3 stages | ND | Yes /No | Positive  (score: 0.93) | No |
| 3 | FLLDFLLYV | 0.998  +/- 1.05E-04 | HLA-A*02 | TGME49_223920  Rhoptry neck protein RON3 | 3 stages | Rhoptry organelles | Yes | Positive  (score: 1.22) | Yes (89%) |
| 4 | FLMDFDFHV | 0.999  +/- 7.86E-05 | HLA-A*02 | TGME49_271335 Hypothetical protein | 3 stages  (Higher in Oocyst) | ND | Yes | Positive  (score: 1.21) | No |
| 5 | FLWPFDYPV | 0.999  +/- 6.88E-05 | HLA-A*02 | TGME49_217480 Hypothetical protein | 3 stages | Nucleus | Yes | Positive  (score: 0.70) | No |
| 6 | YLWHFSYEV | 0.998  +/- 2.81E-04 | HLA-A*02 | TGME49_253290  Valyl-tRNA synthetase | 3 stages | Citosol | Yes /No | Positive  (score: 1.13) | No |
| 7 | MPLGGGFAM | 0.855  +/- 3.75E-03 | HLA-B*35 | TGME49_300130  AMA-1 domain-containing protein | 3 stages  (Higher in Bradyzoite) | Membrane | - | Positive  (score:0.95) | No |
| 8 | FPFAGWTAM | 0.839  +/- 4.23E-03 | HLA-B*35 | TGME49_242020  Mannosyl-oligosaccharide glucosidase | 3 stages | Endoplasmic reticulum | Yes | Positive  (score: 1.10) | No |
| 9 | YPIAPSFAM | 0.813  +/- 1.07E-02 | HLA-B*35 | TGME49_208740  Microneme protein, putative | 3 stages  (Higher in Tachyzoite and bradyzoite) | Micronemes | Yes /No | Positive  (score:0.89) | No |

^A^Expression in the three stages of the parasite: oocyst, tachyzoite, and bradyzoite (RMA>3).

^B^ND: Does not present data in ToxoDB.

^C^ Positive association 🡪 F, I, W, A. Negative association🡪S, K, M, Q (According to literature [49]).

^D^ Exclusion or elimination criteria.

St. 2. 9- and 10-AA peptides selected by neural networks for the HLA-A*24 complex. IC50< 50nM in *Epitope prediction*. Redundant peptide sequences between strains with the highest prediction probabilities for supertype A*24 are shown. Characteristics of the proteins from which the peptides were derived, as determined using ToxoDB and the most important criteria considered in the *in silico* analysis are also presented. All peptides were conserved among the seven *T. gondii* strains analyzed.

| No. | Peptide | Probability  (X̅ +/- SD) | Alleles-affinity | Code Toxo DB Protein | Expression/Stage^A^ | Subcellular Localization ^B^ | TCR- immunogenicity  (presence of AA) ^C^ | Cleavage prediction: proteasome/TAP (score>0,5) ^D^ | BLASTp ^D^  Identity >70% with humans |
| --- | --- | --- | --- | --- | --- | --- | --- | --- | --- |
| 1 | IFLSIYWKI | 0.998  +/- 00E+00 | HLA-A*24 | TGME49_252640  P-type ATPase PMA1 | 3 stages  (Higher in Bradyzoite) | Membrane | Yes/No | Positive  (score: 1.19) | No |
| 2 | VFAFAFFLI | 0.99  +/- 00E+00 | HLA-A*24 | TGME49_273380  Ion channel protein | 3 stages (Higher in Oocyst) | Membrane | Yes | Positive  (Score:0.99) | No |
| 3 | RFAGVFMFF | 0.891 +/- 00E+00 | HLA-A*24 | TGME49_226020  Transporter, major facilitator family protein | 3 stages | Micronemes | Yes | Positive  (score: 1.51) | No |
| 4 | LYLLHSWTW | 0.704  +/- 00E+00 | HLA-A*24 | TGME49_286500  Multidrug resistance efflux transport | 3 stages  (Higher in Tachyzoite) | Membrane | No | Positive  (score: 1.61) | Yes (77%) |
| 5 | RWFWTFRFNW | 0.928 +/- 2.17E-02 | HLA-A*24 | TGME49_239755  Hypothetical protein | 3 stages | Membrane | Yes | SI  (score: 1.24) | No |


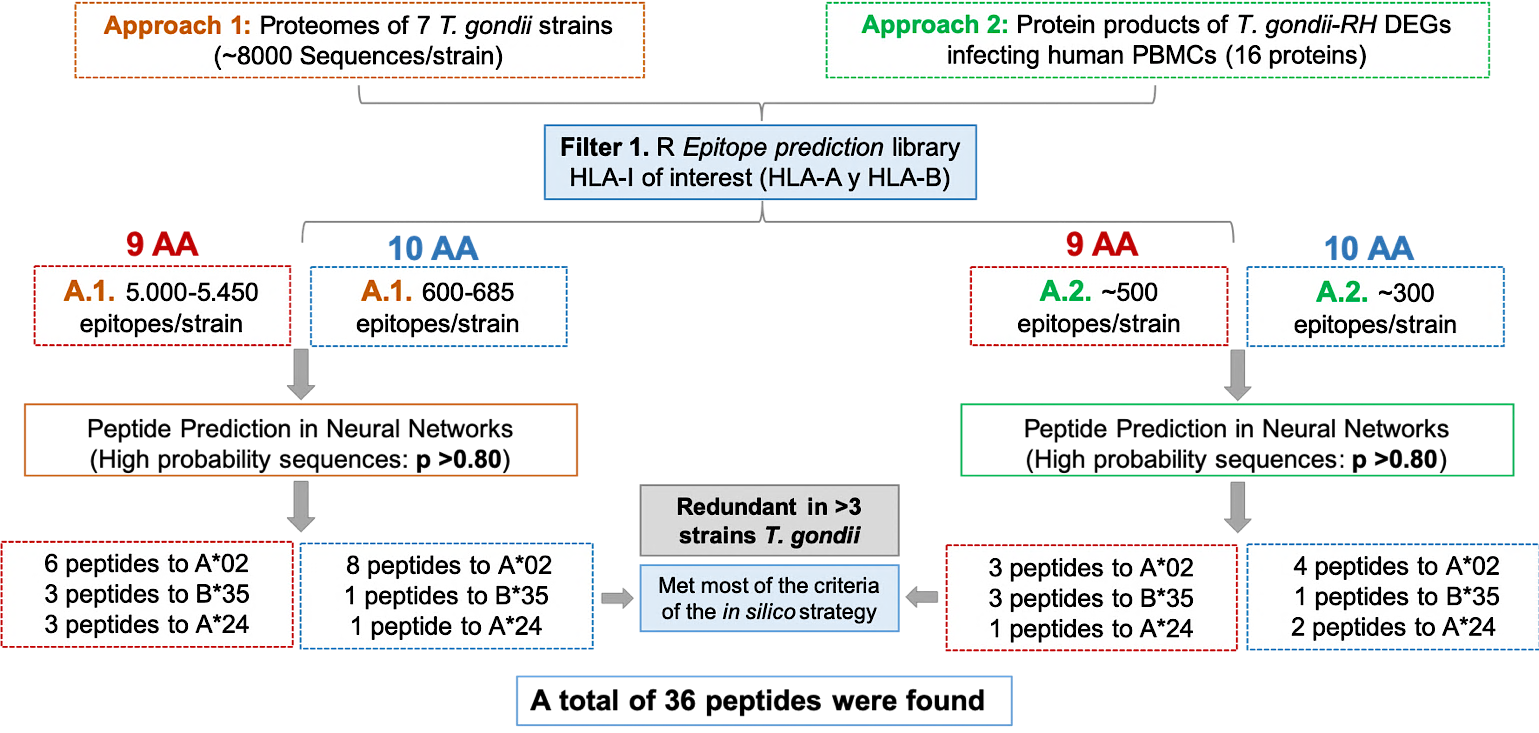


Sf 1. Scheme of the number of *T. gondii* peptides/epitopes identified through in silico analysis. The total numbers of sequences filtered and selected using the two approaches (A1: Approach 1 and A2: Approach 2) are summarized.


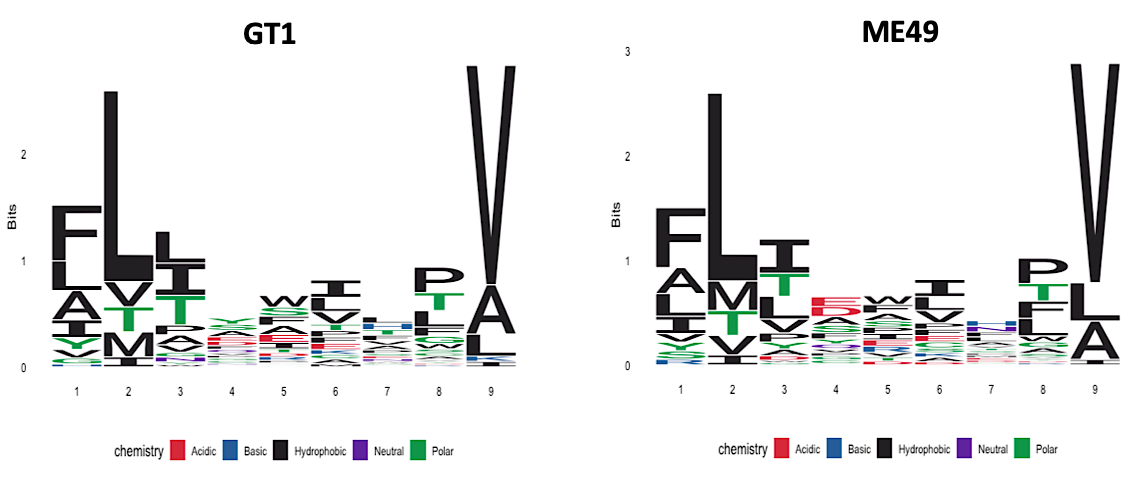


Sf 2. 9 AA peptide logos predicted by the HLA-I network with the highest probability (>0.90) in approach 2. Logos for the strains GT1 and ME49 are shown as examples. Sequence logos were generated using the R *PepTools* library.


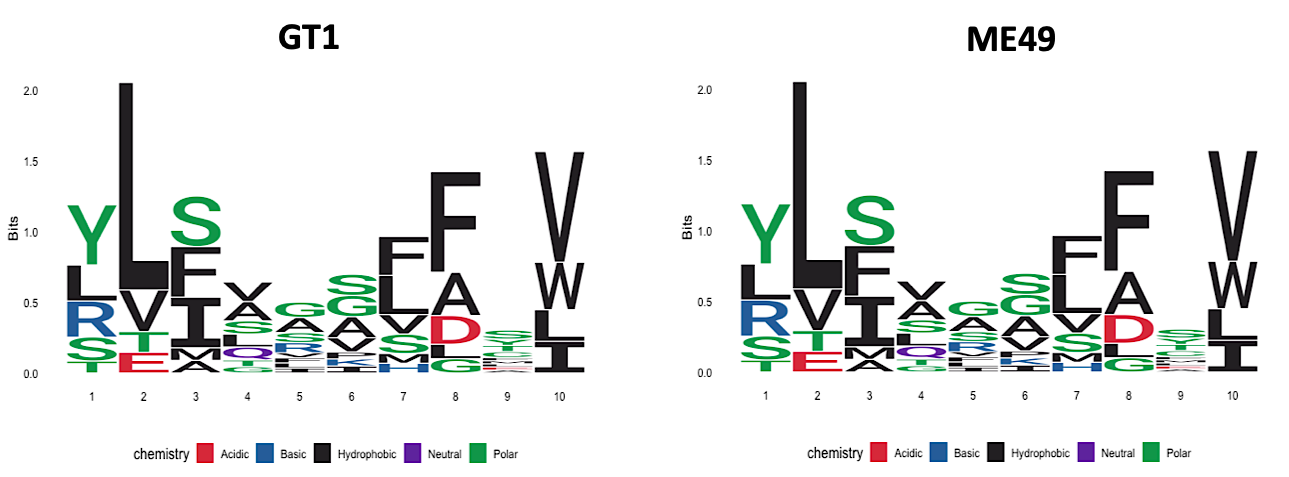


Sf 3. The 10-AA peptide logos predicted by the HLA-I network with the highest probability (>0.85) using Approach 2. Logos for the strains GT1 and ME49 are shown as examples. Sequence logos were generated using the R *PepTools* library.

St 3. 9-AA peptides predicted by neural networks in Approach 2 for HLA-A*02, HLA-B*35, and HLA-A*24 complexes. Redundant peptide sequences between the strains and those with the highest prediction are shown. Characteristics of the proteins from which these peptides were derived, as determined by ToxoDB, and the most important criteria considered in the *in silico* analysis.

| No. | Peptide | Redundancy between strains | Probability  (X̅ +/- SD) | Alleles-affinity | Code Toxo DB and Protein | Expression/Stage^A^ | Subcellular Localization ^B^ | TCR- immunogenicity  (presence of AA) ^C^ | Cleavage prediction: proteasome/TAP (score > 0,5) ^D^ | BLASTp ^D^  Identity >70% with humans |
| --- | --- | --- | --- | --- | --- | --- | --- | --- | --- | --- |
| 1 | ALAEFIETV | 7 | 0.979  +/- 9.47E-03 | HLA-A*02 | TGGT1_252640  P-type ATPase PMA1 | 3 stages  (Higher in Bradyzoite) | Cell membrane | Yes | Positive  (score: 1.06) | No |
| 2 | LLLQWLTTV | 7 | 0.966  +/- 1.88E-02 | HLA-A*02 | TGGT1_253330  Rhoptry kinase family protein | 3 stages  (Higher in Bradyzoite) | Rhoptry organelles | Yes /No | Positive  (score: 0.97) | No |
| 3 | ALLDEILRA | 3 | 0.979 +/- 5.88E-03 | HLA-A*02 | TGGT1_214080 Toxofilin | 3 stages | Cell membrane | Yes | Positive  (score: 1.27) | No |
| 4 | MPVVTTTVL | 7 | 0.804  +/- 1.26E-02 | HLA-B*35 | TGGT1_252640  P-type ATPase PMA1 | 3 stages  (Higher in Bradyzoite) | Cell membrane | - | Positive  (score: 1.67) | No |
| 5 | FPGVIGAPM | 6 | 0.605  +/- 8.28E-02 | HLA-B*35 | TGGT1_266610  Kazal-type protein | 3 stages | ND | Yes | Positive  (score:0.76) | No |
| 6 | FPVLTCDLF | 4 | 0.675  +/- 8.08E-03 | HLA-B*35 | TGGT1_411360  Rhoptry kinase (ROP19A) | 3 stages  (Higher in Tachyzoite) | Rhoptry organelles | - | Positive  (score: 1.08) | No |
| 7 | IFWGILWFF | 7 | 0.590  +/- 5.25E-02 | HLA-A*24 | TGGT1_252640  P-type ATPase PMA1 | 3 stages  (Higher in Bradyzoite) | Cell membrane | Yes | Positive  (score: 1.18) | No |

^A^Expression in the three stages of the parasite: oocyst, tachyzoite, and bradyzoite (RMA>3).

^B^ND: Does not present data in ToxoDB.

^C^ Positive association 🡪 F, I, W, A. Negative association🡪S, K, M, Q (According to the literature [49]).

^D^ Exclusion or elimination criteria.

St 4. 10-AA peptides predicted by neural networks in Approach 2 for HLA-A*02, HLA-B*35, and HLA-A*24 complexes. Redundant peptide sequences between the strains and those with the highest prediction are shown. Characteristics of the proteins from which these peptides were derived, as determined by ToxoDB, and the most important criteria considered in the *in silico* analysis.

| No. | Peptide | Redundancy between strains | Probability  (X̅ +/- SD) | Alleles-affinity | Code Toxo DB and Protein | Expression/Stage^A^ | Subcellular Localization ^B^ | TCR- immunogenicity  (presence of AA) ^C^ | Cleavage prediction: proteasome/TAP (score > 0,5) ^D^ | BLASTp ^D^  Identity >70% with humans |
| --- | --- | --- | --- | --- | --- | --- | --- | --- | --- | --- |
| 1 | YLIALKHAFI | 7 | 0.957  +/- 1.17E-02 | HLA-A*02 | TGGT1_252640  P-type ATPase PMA1 | 3 stages  (Higher in Bradyzoite) | Cell membrane | Yes | Positive  (score: 1.19) | No |
| 2 | LLIVAALFSV | 7 | 0.900  +/- 5.31E-02 | HLA-A*02 | TGGT1_252640  P-type ATPase PMA1 | 3 stages  (Higher in Bradyzoite) | Cell membrane | Yes | Positive  (score: 0.95) | No |
| 3 | YLSQSVSFCV | 6 | 0.737  +/- 1.61 E-01 | HLA-A*02 | TGGT1_278090  Toxoplasma gondii family A protein | 3 stages  (Higher in Bradyzoite) | ND | No | Positive  (score: 1.07) | No |
| 4 | SLFSGGVFTL | 5 | 0.810  +/- 1.50 E-03 | HLA-A*02 | TGGT1_411360  Rhoptry kinase family protein | 3 stages  (Higher in Tachyzoite) | Rhoptry organelles | No | Positive  (score: 1.55) | No |
| 5 | FPLGSRFSPF | 6 | 0.636  +/- 6.13E-02 | HLA-B*35 | TGGT1_249150  PAN domain-containing protein | 3 stages | ND | No | Positive  (score: 0.75) | No |
| 6 | VWILALLIMF | 5 | 0.518  +/- 1.70E-01 | HLA-A*24 | TGGT1_219348  SAG-related sequence SRS55M | 3 stages | Cell membrane | Yes | Positive  (score:1.40) | No |
| 7 | RYSTFRPALF | 4 | 0.510  +/- 2.06E-02 | HLA-A*24 | TGGT1_207140  SAG-related sequence SRS49B | 3 stages  (Higher in Bradyzoite) | Cell membrane | Yes | Positive  (score: 1.13) | No |

^A^Expression in the three stages of the parasite: oocyst, tachyzoite, and bradyzoite (RMA>3).

^B^ND: Does not present data in ToxoDB.

^C^ Positive association 🡪 F, I, W, A. Negative association🡪S, K, M, Q (According to the literature [49]).

^D^ Exclusion or elimination criteria.

**St 5.** Serological test results of 50 individuals included in the study. The results of PCRs for HLA-I of interest are also shown in the table. ^A^Values equal to or greater than 8 IU/ml of IgG indicate positivity for the serological test. ^B^ Values equal to or greater than 0.65 IU/ml of IgM indicate positivity of the serological test.

| Samples  CODE | IgG Anti-*T. gondii*  (UI/ml) ^A^ | IgM Anti-*T. gondii*  (UI/ml) ^B^ | A*02 | HLA-I - PCR  A*24 | B*35 |
| --- | --- | --- | --- | --- | --- |
| 1 | 0 | 0.03 | POSITIVE | NEGATIVE | NEGATIVE |
| 2 | >300 | 0.05 | POSITIVE | POSITIVE | NEGATIVE |
| 3 | 0 | 0.07 | NEGATIVE | POSITIVE | NEGATIVE |
| 4 | 7 | 0.06 | POSITIVE | NEGATIVE | NEGATIVE |
| 5 | 0 | 0.05 | POSITIVE | NEGATIVE | NEGATIVE |
| 6 | 38 | 0.07 | POSITIVE | POSITIVE | NEGATIVE |
| 7 | 0 | 0.04 | NEGATIVE | NEGATVE | NEGATIVE |
| 8 | 163 | 0.25 | POSITIVE | NEGATIVE | POSITIVE |
| 9 | 0 | 0.5 | POSITIVE | POSITIVE | NEGATIVE |
| 10 | 28 | 0.04 | NEGATIVE | POSITIVE | POSITIVE |
| 11 | 10 | 0.11 | NEGATIVE | POSITIVE | NEGATIVE |
| 12 | 170 | 0.39 | NEGATIVE | POSITIVE | NEGATIVE |
| 13 | 0 | 0.03 | NEGATIVE | POSITIVE | POSITIVE |
| 14 | 0 | 0.03 | NEGATIVE | NEGATIVE | NEGATIVE |
| 15 | 0 | 0.14 | NEGATIVE | POSITIVE | NEGATIVE |
| 16 | 0 | 0.06 | NEGATIVE | POSITIVE | POSITIVE |
| 17 | 0 | 0.05 | NEGATIVE | POSITIVE | NEGATIVE |
| 18 | 0 | 0.1 | NEGATIVE | POSITIVE | POSITIVE |
| 19 | 0 | 0.07 | POSITIVE | POSITIVE | NEGATIVE |
| 20 | 69 | 0.04 | NEGATIVE | POSITIVE | NEGATIVE |
| 21 | 0 | 0.04 | NEGATIVE | NEGATIVE | POSITIVE |
| 22 | >300 | 0.14 | POSITIVE | NEGATIVE | NEGATIVE |
| 23 | 77 | 0.08 | POSITIVE | POSITIVE | NEGATIVE |
| 24 | 0 | 0.03 | NEGATIVE | POSITIVE | NEGATIVE |
| 25 | 0 | 0.03 | POSITIVE | NEGATIVE | NEGATIVE |
| 26 | 0 | 0.03 | NEGATIVE | POSITIVE | POSITIVE |
| 27 | 0 | 0.03 | POSITIVE | NEGATIVE | POSITIVE |
| 28 | 0 | 0.03 | NEGATIVE | POSITIVE | NEGATIVE |
| 29 | 59 | 0.02 | POSITVE | NEGATIVE | NEGATIVE |
| 30 | 48 | 0.03 | NEGATIVE | POSITIVE | NEGATIVE |
| 31 | 0 | 0.07 | NEGATIVE | POSITIVE | NEGATIVE |
| 32 | 0 | 0.05 | POSITIVE | POSITIVE | NEGATIVE |
| 33 | 0 | 0.14 | NEGATIVE | NEGATIVE | NEGATIVE |
| 34 | 0 | 0.05 | NEGATIVE | POSITIVE | POSITIVE |
| 35 | 12 | 0.04 | POSITIVE | NEGATIVE | NEGATIVE |
| 36 | 0 | 0.03 | NEGATIVE | POSITIVE | NEGATIVE |
| 37 | 62 | 0.05 | NEGATIVE | POSITIVE | NEGATIVE |
| 38 | 110 | 0.23 | NEGATIVE | POSITIVE | NEGATIVE |
| 39 | 0 | 0.04 | POSITIVE | NEGATIVE | NEGATIVE |
| 40 | 69 | 0.05 | NEGATIVE | NEGATIVE | NEGATIVE |
| 41 | 0 | 0.03 | POSITIVE | POSITIVE | NEGATIVE |
| 42 | 0 | 0.04 | NEGATIVE | POSITIVE | NEGATIVE |
| 43 | 151 | 0.21 | NEGATIVE | POSITIVE | NEGATIVE |
| 44 | 0 | 0.06 | NEGATIVE | POSITIVE | NEGATIVE |
| 45 | 176 | 0.05 | NEGATIVE | POSITIVE | NEGATIVE |
| 46 | 123 | 0.05 | NEGATIVE | POSITIVE | POSITIVE |
| 47 | 226 | 0.08 | POSITIVE | POSITIVE | NEGATIVE |
| 48 | 0 | 0.04 | NEGATIVE | NEGATIVE | NEGATIVE |
| 49 | 0 | 0.02 | POSITIVE | POSITIVE | POSITIVE |
| 50 | 34 | 0.05 | NEGATIVE | POSITIVE | NEGATIVE |

**St6.** Peptides selected by *in silico* analysis, synthesized, and evaluated by ELISPOT *ex vivo* assays to measure IFN-γ response. Peptides restricted to HLA-A*02 are shown in orange, those corresponding to HLA-A*24 are in red, and those predicted for HLA-B*35 are in purple. A1: Approach 1and A2: Approach 2.

| **Final**  **Code** | **Peptide** | **Protein name** | **Location (AA)** | **Allele** | **Approach** |
| --- | --- | --- | --- | --- | --- |
| **P1** | **FLFAWITYV** | DHHC3 palmitoyl transferase | 22 | HLA-A*02 | A.1 |
| P2 | FLWPFDYPV | Hypothetical protein | 807 | HLA-A*02 | A.1 |
| P3 | YLWHFSYEV | Valyl-tRNA synthetase | 299 | HLA-A*02 | A.1 |
| P10 | ALAEFIETV | P-type ATPase PMA1 | 664 | HLA-A*02 | A.2 |
| P11 | LLLQWLTTV | Rhoptry kinase family protein | 15 | HLA-A*02 | A.2 |
| P12 | ALLDEILRA | Toxofilin | 109 | HLA-A*02 | A.2 |
| P19 | FLVDGTFFSV | Hypothetical protein | 1627 | HLA-A*02 | A.1 |
| P23 | LLIVAALFSV | P-type ATPase PMA1 | 183 | HLA-A*02 | A.2 |
| P24 | YLSQSVSFCV | *Toxoplasma gondii* family A protein | 160 | HLA-A*02 | A.2 |
| P25 | SLFSGGVFTL | Rhoptry kinase family protein (ROP19A) | 226 | HLA-A*02 | A.2 |
| P7 | IFLSIYWKI | P-type ATPase PMA1 | 931 | HLA-A*24 | A.1 |
| **P8** | **VFAFAFFLI** | Ion channel protein | 193 | HLA-A*24 | A.1 |
| P9 | RFAGVFMFF | Transporter, major facilitator family protein | 433 | HLA-A*24 | A.1 |
| P21 | VYFMSASYF | Ubiquinol cytochrome c oxidoreductase | 326 | HLA-A*24 | A.2 |
| P22 | RWFWTFRFNW | Putative transmembrane protein | 15 | HLA-A*24 | A.1 |
| P27 | RYSTFRPALF | SAG-related sequence SRS49B | 12 | HLA-A*24 | A.2 |
| P4 | MPLGGGFAM | AMA-1 domain-containing protein | 136 | HLA-B*35 | A.1 |
| P5 | FPFAGWTAM | Mannosyl-oligosaccharide glucosidase | 1367 | HLA-B*35 | A.1 |
| **P6** | **YPIAPSFAM** | Microneme protein, putative | 21 | HLA-B*35 | A.1 |
| P13 | MPVVTTTVL | P-type ATPase PMA1 | 406 | HLA-B*35 | A.2 |
| P14 | FPGVIGAPM | Kazal-type serine protease inhibitor domain-containing protein | 526 | HLA-B*35 | A.2 |
| P15 | FPVLTCDLF | Rhoptry kinase family protein (ROP19A) | 353 | HLA-B*35 | A.2 |
| P20 | FPIQEVDAPF | Histone methylation protein DOT1 | 1930 | HLA-B*35 | A.1 |
| P26 | FPLGSRFSPF | PAN domain-containing protein | 1475 | HLA-B*35 | A.2 |


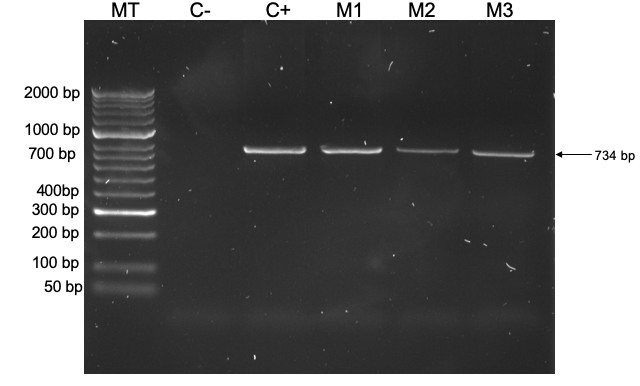


Sf 4. Electrophoresis was used to visualize the HLA-A*02 allele, with an expected band of approximately 734 bp. MT: Hyperladder II molecular size marker from 50 bp to 2000 bp (BIOLINE). C-: negative control (distilled water); C+: positive control (sample positive for HLA-A*02 and confirmed by sequencing in a previous study). M1-M3: DNA samples from 3 individuals included in the study.


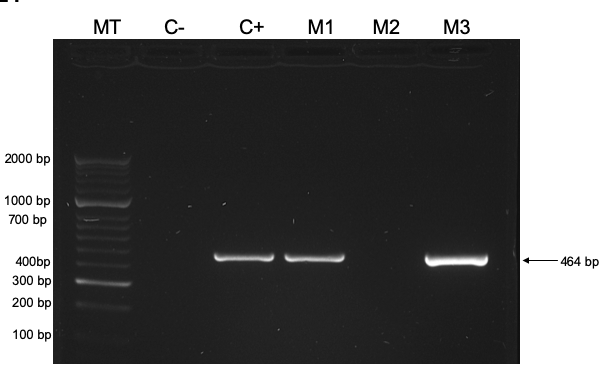


Sf 5. Electrophoresis was used to visualize the HLA-A*24 supertype with an expected band of approximately 464 bp. MT: Hyperladder II molecular size marker from 50 bp to 2000 bp (BIOLINE). C-: negative control (distilled water); C+: positive control (HLA-A*24 positive sample). M1-M3: DNA samples from 3 individuals included in the study.


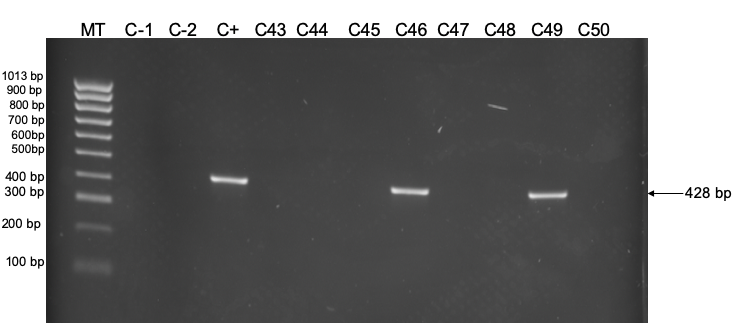


Sf 6. Electrophoresis was used to visualize the HLA-B*35 allele with an expected band of approximately 428 bp. MT: HyperLadder™ 100bp molecular size marker (BIOLINE). C-1: negative control (distilled water). C-2: Negative control (DNA sample negative for the allele). C+: Positive control (sample positive for HLA-B*35). C43-C50: DNA samples from 8 individuals included in this study.


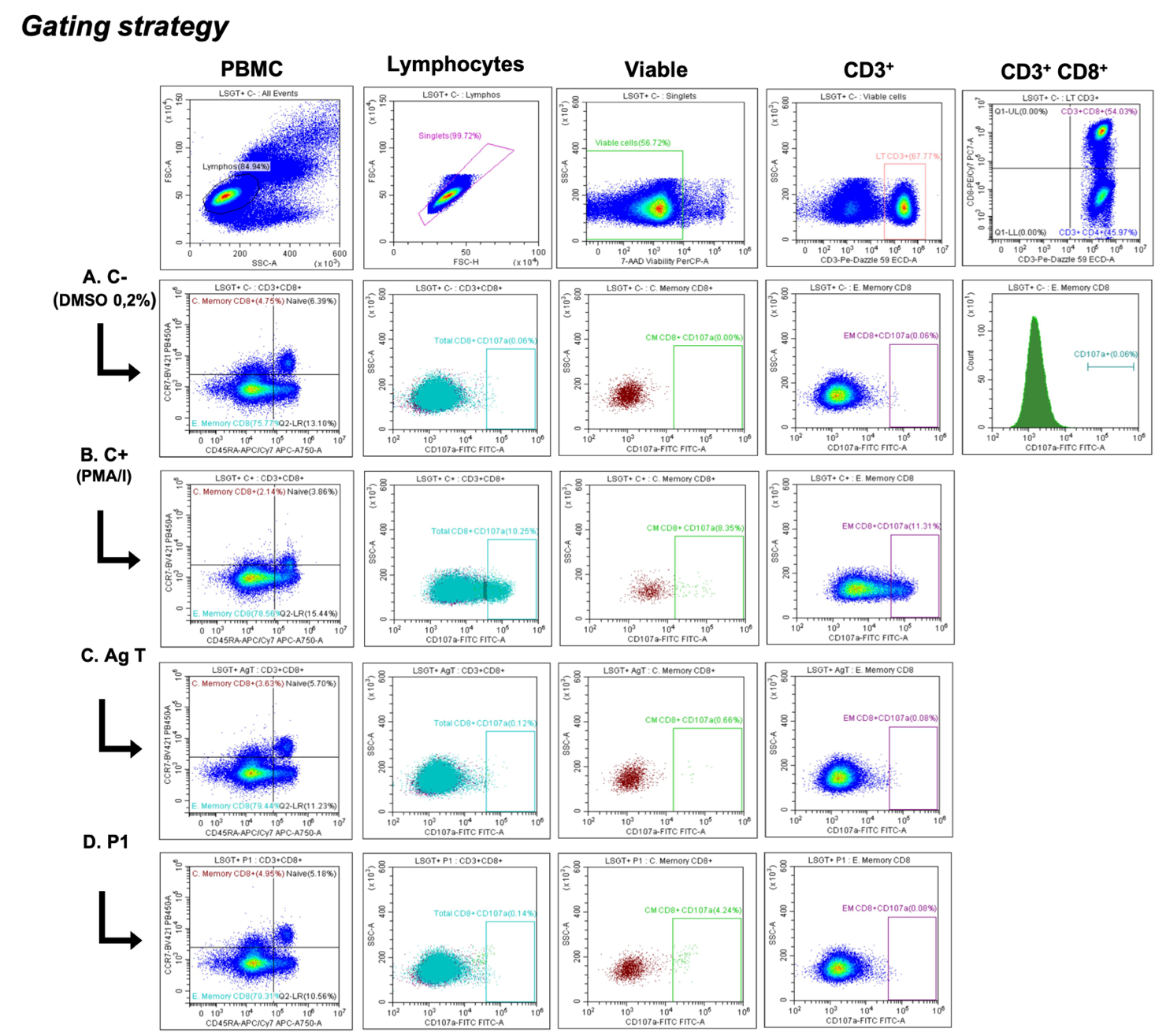


Sf7. Representative gating strategy for detecting the degranulation marker CD107a in PBMC from *T. gondii* seropositive and HLA-A*02+ individuals after 6 h of stimulation. The dot plots in the upper panel represent the gates performed for the viable CD3+CD8+ lymphocyte population, as described in the Methods section. (A) Representation of % CD3+CD8+ expressing CD107a+ (turquoise blue box), central memory (CM, green box), and effector (EM, purple box) stimulated with negative control (C-, DMSO 0.2%), (B) positive control (C+) with PMA/I, (C) with T. gondii total antigen (Ag T), and (D) with T. gondii P1 peptide.
